# Copper-instigated modulatory cell mortality mechanisms and progress in oncological treatment investigations

**DOI:** 10.3389/fimmu.2023.1236063

**Published:** 2023-08-02

**Authors:** Lei Gao, Anqi Zhang

**Affiliations:** ^1^ Medical Imaging Department, Huabei Petroleum Administration Bureau General Hospital, Renqiu, China; ^2^ Oncology Department, Huabei Petroleum Administration Bureau General Hospital, Renqiu, China

**Keywords:** copper, cuprotosis, regulatory cell death, copper complexes, antitumor activity

## Abstract

Copper, a transition metal, serves as an essential co-factor in numerous enzymatic active sites and constitutes a vital trace element in the human body, participating in crucial life-sustaining activities such as energy metabolism, antioxidation, coagulation, neurotransmitter synthesis, iron metabolism, and tetramer deposition. Maintaining the equilibrium of copper ions within biological systems is of paramount importance in the prevention of atherosclerosis and associated cardiovascular diseases. Copper induces cellular demise through diverse mechanisms, encompassing reactive oxygen species responses, apoptosis, necrosis, pyroptosis, and mitochondrial dysfunction. Recent research has identified and dubbed a novel regulatory cell death modality—”cuprotosis”—wherein copper ions bind to acylated proteins in the tricarboxylic acid cycle of mitochondrial respiration, resulting in protein aggregation, subsequent downregulation of iron-sulfur cluster protein expression, induction of proteotoxic stress, and eventual cell death. Scholars have synthesized copper complexes by combining copper ions with various ligands, exploring their significance and applications in cancer therapy. This review comprehensively examines the multiple pathways of copper metabolism, copper-induced regulatory cell death, and the current status of copper complexes in cancer treatment.

## 1 Introduction

Copper, an essential trace element in the human body, functions as a vital co-factor for numerous enzymes within living organisms, boasting robust redox activity and protein-binding capabilities. Through the maintenance and regulation of intracellular copper homeostasis, it partakes in modulating cellular physiological functions. Exposure to external environmental factors may trigger imbalances in cellular copper metabolism, mediating cytotoxic effects and bodily damage (1, 2). It is well-established that the regulation of regulated cell death (RCD) plays a pivotal role in determining cellular fate (3, 4); however, the mechanisms underlying copper-induced cytotoxicity and cell death have not yet been fully elucidated. Recently, Tsvetkov and colleagues, in a research article published in Science, demonstrated the existence of a copper-dependent, regulated cell death in human cells that relies on mitochondrial respiration but is distinct from known cell death mechanisms, such as apoptosis, necrotic cell death, pyroptosis, and ferroptosis; this novel form of RCD has been termed “cuprotosis” (5). Although cuprotosis has not yet been officially recognized in the nomenclature of cell death, evidence suggests that the mechanisms of copper-induced cell death involve not only intracellular copper accumulation but also share common markers and features with different forms of RCD, such as reactive oxygen species generation, apoptosis, necrosis, pyroptosis, and mitochondrial dysfunction. This review primarily focuses on ketone metabolism, the various pathways of copper-induced regulatory cell death, and the current status of copper complexes in cancer treatment.

## 2 Metabolism, transport, and the relationship between copper ions and cancer within the body

Copper, an indispensable trace metal element in humans, is involved in energy production, iron metabolism, neuropeptide activation, connective tissue synthesis, and neurotransmitter synthesis (6). As a cofactor for numerous essential enzymes (cuproenzymes), copper constitutes the body’s defense system, enhancing cellular anti-inflammatory and antioxidative capabilities, and strengthening immune functions (7). On one hand, the maintenance and regulation of copper homeostasis is crucial for cellular physiological functions due to its robust redox activity and protein-binding capacity (8). On the other hand, copper plays a unique role in maintaining and activating the immune system (4). It is vital for the normal growth, development, and maintenance of bones, connective tissues, the brain, the heart, and various other organs. Copper ions promote lymphocyte proliferation and activation and serve as structural components of serum immunoglobulins, playing a crucial role in the transformation of IgM to IgG. Moreover, copper ions influence the immune functions of the body by modulating the levels of tumor necrosis factors, interferons, IL-2, and other factors, thereby affecting the development and activity regulation of T cells, B cells, natural killer cells, and macrophages (9, 10).

**Figure 1:**
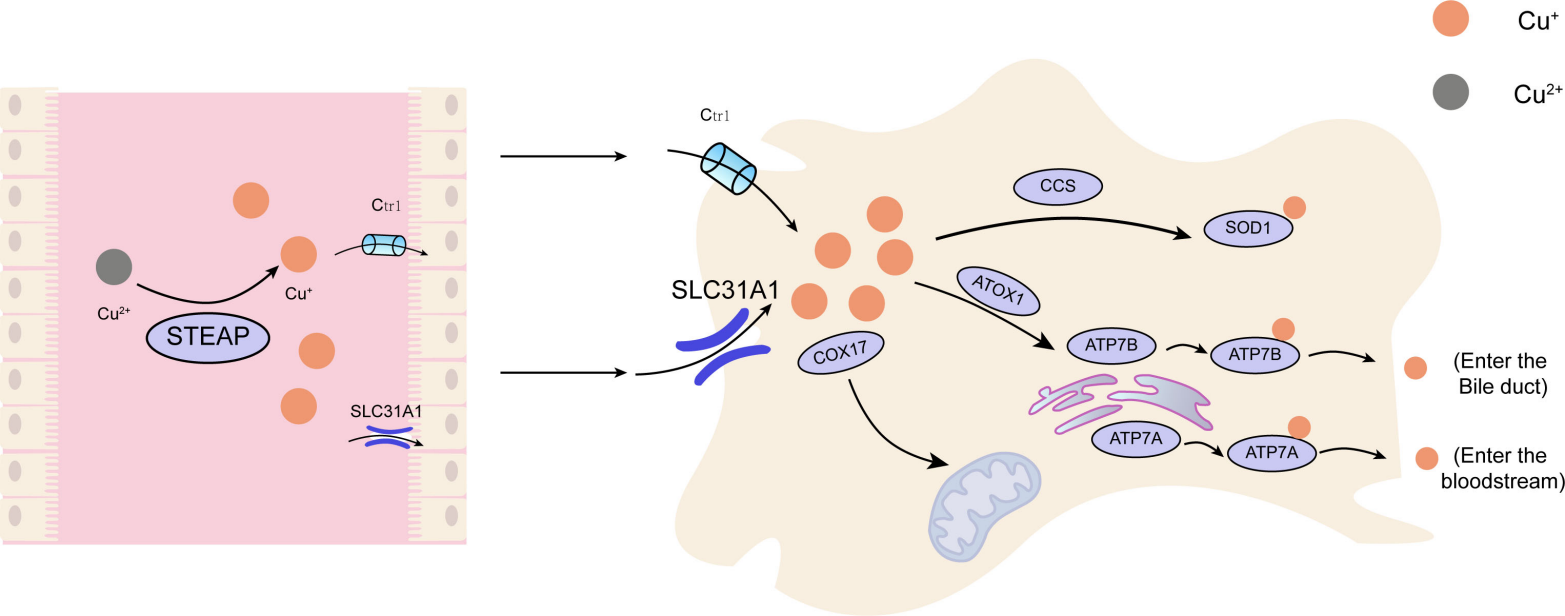
The principal metabolic pathway of copper within cellular structures. Cu, copper; Ctr1, copper transporter 1；CCS, copper chaperone for superoxide dismutase;SOD1, superoxide dismutase 1; COX17, cytochrome c oxidase copper chaperone 17; SLC31A1,Human High affinity copper uptake protein 1; ATOX1, antioxidant protein 1; ATP7A, ATPdependentcopper transporter 7A; ATP7B, ATP-dependent copper transporter 7B.

**Figure 2:**
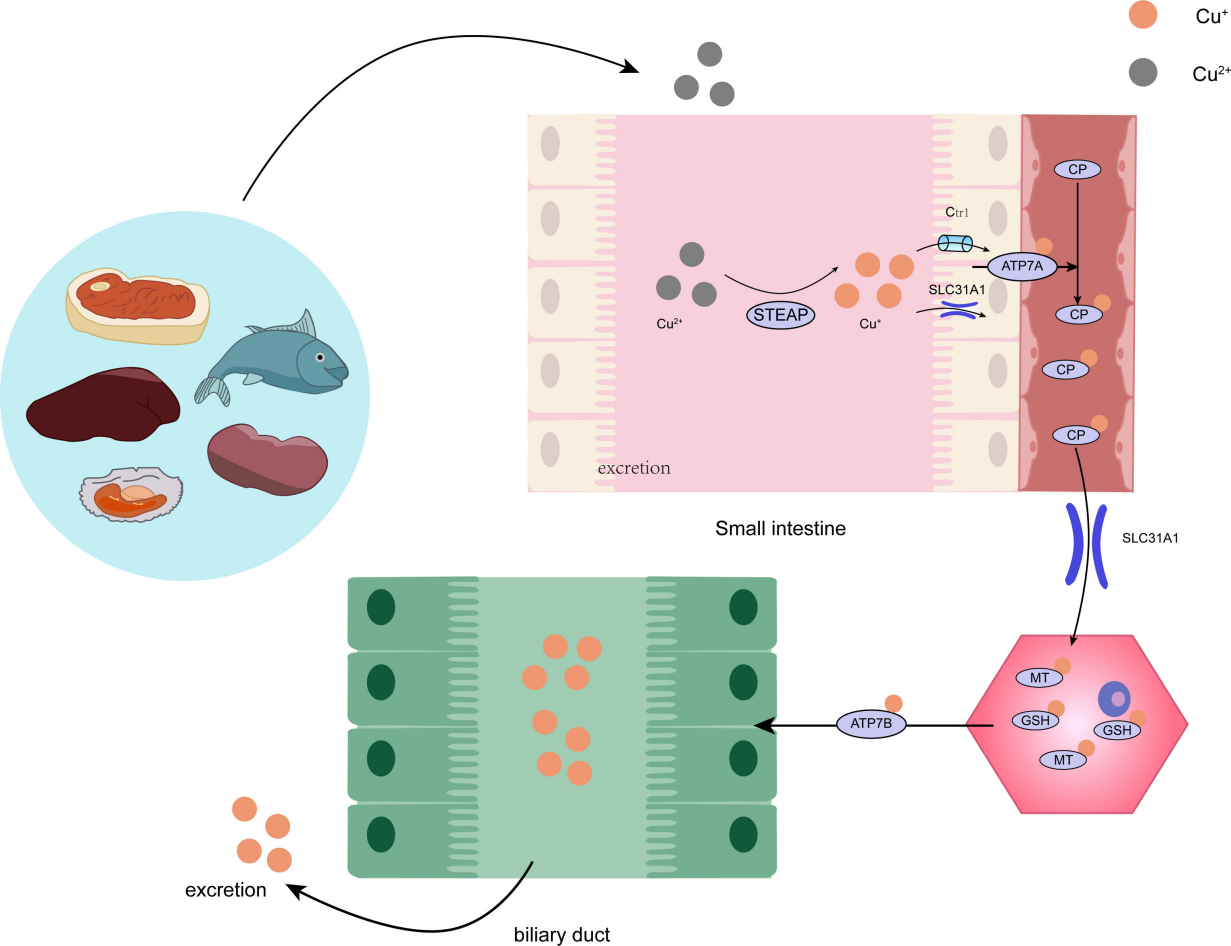
The intricate procedure of copper metabolism within the human organism. STEAP, STEAP Protein Family; CP, ceruloplasmin; SLC31A1, Human High affinity copperuptake protein 1; GSH, glutathione; MT, metallothionein.

Copper is found in various plants and animal foods, with men consuming approximately 1,400 micrograms and women 1,100 micrograms daily through their diets. The current recommended copper intake for adults is 0.8-2.4 mg/day to maintain systemic copper homeostasis. The absorption, transport, storage, and excretion of copper within organisms and cells determines its distribution and homeostatic regulation. Copper transport in the body involves numerous copper chaperone and transport proteins, including CTR1, Steap proteins, cytochrome c oxidase copper chaperone 17 (COX17), antioxidant protein (Atox), copper chaperone for superoxide dismutase (CCS), and superoxide dismutase 1 (SOD1). Extracellular copper primarily exists as Cu^2+^, which is reduced to Cu^+^ by metal reductases (such as the Steap family) on the cell surface before entering the cell. Cu^+^ is mainly absorbed in the intestines, with copper transporter 1 (CTR1) existing as a homotrimer on the intestinal epithelial cell membrane, displaying high specificity for Cu^+^ uptake and subsequent cellular entry (11). Once inside the cell, copper follows three primary distribution pathways: COX17 transports cytoplasmic copper ions to the mitochondrial intermembrane space and further delivers them to the cysteine residues of SCO1, forming disulfide bonds and participating in the assembly of cytochrome c oxidase copper ions within the mitochondria (12). Atox1 mediates copper transmembrane secretion, maintaining intracellular copper ion homeostasis and function. Atox1, carrying copper ions, is transported to the Golgi apparatus, where it binds to ATP-dependent copper transporter 7A (ATP7A) and ATP-dependent copper transporter 7B (ATP7B) on the trans-Golgi network. ATP7A and ATP7B are associated with extracellular copper ion secretion (13, 14). CCS mediates the movement of copper to SOD1, an antioxidant metalloenzyme crucial for maintaining the balance between oxidation and antioxidation in organisms (15, 16). SOD1 catalyzes the conversion of superoxide radicals into hydrogen peroxide and maintains the steady state of reactive oxygen species (ROS) within cells (**Figure 1**). Ultimately, these copper proteins, assembled with copper ions, are sorted into specific organelles or transported to the portal vein via the Atox1/ATPase (ATP7A) pathway. Intestinal Cu^+^ absorption enters the peripheral circulation and is transported to the liver via the portal vein system. Copper circulates systemically, bound to plasma proteins such as ceruloplasmin (CP), albumin, and transcuprein (17). Ceruloplasmin is the primary copper carrier protein in human plasma, accounting for 75% of total plasma copper. The liver serves as the main storage and excretory organ for copper (18). Copper stored in hepatocytes is either released into the bloodstream for further action or transported into bile for excretion. ATP7A facilitates copper entry into brain tissue across the blood-brain barrier, while ATP7B regulates intracellular copper excretion. When copper levels are excessive, Cu^+^ in hepatocytes binds to the copper chaperone antioxidant protein 1, which associates with the N-terminal metal-binding domain of ATP7B and transports copper to the bile canaliculus membrane for excretion (**Figure 2**) (19). Maintaining appropriate copper intake is crucial for normal biological functions. Copper deficiency may result in anemia, skeletal disorders, neurological dysfunction, and immune system damage. Excessive copper intake can lead to liver damage, gastrointestinal symptoms, and kidney injury (20).

In fact, the connection between copper and cancer has long attracted the attention of researchers, with numerous studies indicating that tumor tissues require higher levels of copper compared to healthy tissues. Balanced gastrointestinal absorption and biliary excretion are essential for maintaining copper homeostasis in the human body during normal growth and development (21, 22). Many studies have shown increased copper concentrations in tumors or serum, including breast, lung, gastrointestinal, oral, thyroid, gallbladder, gynecological, and prostate cancers (23, 24). Copper imbalance not only affects mitochondrial respiration but also leads to alterations in glycolysis, insulin resistance, and lipid metabolism. Moreover, copper regulates autophagy through ULK1 and ULK2 and/or controls protein quality through UBE2D2, providing novel copper-dependent targets that can influence tumor growth and progression (2). Copper also promotes tumor angiogenesis, contributing to tumor initiation, growth, and metastasis (25). This metal nutrient directly activates various angiogenic factors, including vascular endothelial growth factor (VEGF), fibroblast growth factor 2 (FGF2), tumor necrosis factor (TNF), and interleukin-1 (IL-1) (26, 27).

A considerable amount of research has noted significantly elevated copper levels in tumor tissues and serum of a range of cancers, such as melanoma (28), breast (29), prostate (30), liver (31), lung (32), and gastric cancers (33). Clinical studies on patients with hepatolenticular degeneration have shown that abnormal copper accumulation in the body can induce malignant transformation of hepatocytes. Therefore, copper ion levels are closely associated with the initiation and development of tumors.

Copper imbalance not only affects mitochondrial respiration but also leads to changes in glycolysis, insulin resistance, and lipid metabolism. In addition to mitochondrial function, copper pathways, such as the ATOX-ATP7A-LOX pathway, promote metastatic expansion (34). Furthermore, copper provides new copper-dependent targets by regulating autophagy and/or controlling protein quality through UBE2D2, affecting tumor growth and progression (2). In the mitogen-activated protein kinase (MAPK) signaling pathway, copper ions can directly bind to MEK1, promoting ERK 1/2 phosphorylation and subsequently activating downstream c-Jun N-terminal kinase (JNK) to regulate tumor growth (35, 36). Recent research has found that copper ions can bind to 3-phosphoinositide-dependent protein kinase-1 (PDK1), promoting its interaction with the Ser/Thr protein kinase AKT (also known as protein kinase B) and activating AKT’s oncogenic signaling in a phosphatidylinositol-3-kinase (PI3K)-dependent manner (22, 37). Inhibiting the copper axis can reduce AKT signaling and suppress tumor initiation and progression, suggesting a close relationship between the PI3K-PDK1-AKT axis and tumor proliferation.

Copper also plays a crucial role in promoting tumor angiogenesis, contributing to tumor initiation, growth, and metastasis. It serves as a vital cofactor in angiogenic signaling cascades. Hypoxic conditions within tumors stimulate angiogenesis by upregulating hypoxia-inducible factor-1α (HIF-1α), which in turn induces the production of vascular endothelial growth factor (VEGF). The strong interaction between HIF-1α and VEGF rapidly activates angiogenic cascades, accelerating tumor angiogenesis (38). HIF-1α is the primary transcription factor regulating VEGF expression. Copper ions are required for the interaction between HIF-1α and target gene hypoxia response elements, ensuring the formation of the HIF-1α transcription complex and activating the expression of target genes, including VEGF (39). Excessive copper ions stabilize the conformation of HIF-1α, promoting its nuclear accumulation and activation, thereby intensifying tissue hypoxia. Furthermore, the massive release of copper directly activates various angiogenic factors, including VEGF, fibroblast growth factor 2 (FGF2), tumor necrosis factor (TNF), and interleukin-1 (IL-1), promoting inflammatory crosstalk between tumor cells and tumor-associated macrophages, thereby stimulating local angiogenesis (40). The significant role of copper in promoting angiogenesis has made copper chelators effective angiogenesis inhibitors in cancer therapy.

## 3 Mechanism of copper-induced regulated cell death

Regulated cell death (RCD), also known as programmed cell death, is a highly controlled process that occurs in multicellular organisms. It is essential for maintaining cellular homeostasis and tissue development and function and can serve as a defense mechanism against infections, mutations, and other cellular damages. Regulated cell death includes three major categories: apoptosis, autophagy, and necrosis. The regulation of RCD is crucial in determining cell fate. Recently, Tsvetkov and colleagues, in a research paper published in Science, confirmed the existence of a copper-dependent, regulated cell death in human cells that depends on mitochondrial respiration but differs from known cell death mechanisms (such as apoptosis, necroptosis, pyroptosis, and ferroptosis) as a novel RCD mode; this new copper-dependent cell death mode has been named “cuprotosis” (5). “Cuprotosis”, copper ions bind to acylated proteins in the tricarboxylic acid cycle of mitochondrial respiration, leading to the aggregation of acylated protein modifications, which in turn downregulate iron-sulfur cluster protein expression, inducing protein toxicity stress and ultimately resulting in cell death.

### 3.1 Copper-induced apoptosis

Apoptosis, a genetically controlled and orderly form of cell death, serves to maintain intracellular stability. It can be triggered by disturbances in the cell’s internal environment, such as DNA replication errors or damage, endoplasmic reticulum stress, and reactive oxygen species overload, or by external stimuli (typically activated by death receptors or dependence receptors), leading to either endogenous or exogenous apoptosis. This process regulates cellular physiological functions and modulates various injury and disease-related pathological processes (41, 42). The Fenton reaction, a metal-mediated reaction, generates hydroxyl radicals (•OH) from hydrogen peroxide (H_2_O_2_) and metal ions (43). In mitochondria, Cu^2+^-MPP complexes induce the Fenton reaction with endogenous H_2_O_2_, generating highly reactive •OH radicals that form oxidative stress, which can react with DNA and lipids, causing DNA damage and lipid peroxidation, harming cells and DNA (44). The Haber-Weiss reaction involves the interaction of hydrogen peroxide and superoxide radicals that produce hydroxyl radicals. The toxicity of H_2_O_2_ primarily stems from its conversion into hydroxyl radicals (•OH) through ionizing radiation (as shown in reaction a), interaction with copper via Fenton chemistry (as shown in reaction b), or interaction with superoxide anion radicals via the Haber-Weiss reaction (as shown in reaction c). Superoxide radicals, through the combination of reactions (d), lead to the cycling of copper between its oxidized and reduced states, resulting in the formation of hydroxyl radicals (•OH) at a considerable rate. Copper may generate an excess of hydroxyl radicals (•OH) by shuttling between Cu^+^ and Cu^2+^ states. The continuous production of hydroxyl radicals (•OH) is toxic to cells, damaging crucial biomolecules such as proteins, lipids, and nucleic acids and disrupting iron-sulfur clusters (45). This damage can trigger apoptosis by activating various signaling pathways, such as the mitogen-activated protein kinase (MAPK) (46) and c-Jun N-terminal kinase (JNK) pathways (47). Simultaneously, it can cause mitochondrial membrane depolarization, cell cycle arrest, and the induction of apoptosis (48).

Reaction a: H_2_O_2_ → 2(•OH)
Reaction b: H_2_O_2_ + Cu^+^ → Cu^2+^ + •OH + ^-^OH
Reaction c: O^•^_2_^-^ + H_2_O_2_ → O_2_ + •OH + ^-^OH
Reaction d: Cu^2+^ + O^•^_2_^-^ → Cu^+^ + O_2_
(Reaction a: H_2_O_2_ → 2(•OH)
Reaction b: H_2_O_2_ + Cu^+^ → Cu^2+^ + •OH + ^-^OH
Reaction c: O^•^_2_^-^ + H_2_O_2_ → O_2_ + •OH + ^-^OH
Reaction d: Cu^2+^ + O^•^_2_^-^ → Cu^+^ + O_2_

Reactive oxygen species (ROS) production can damage biomolecules, including DNA and chromatin, leading to the activation of DNA repair mechanisms and cell cycle checkpoints such as the tumor suppressor protein p53. Copper can interact with p53, playing a crucial role in regulating cell cycle progression and apoptosis (49). Formigari et al. indicate that intracellular free zinc modulates p53 activity and stability, with excess zinc altering p53 protein structure and downregulating its binding to DNA. Copper can also displace zinc from its normal binding site on p53, resulting in aberrant protein folding and p53 function disruption (50). p53 activation appears to play a pivotal role in copper and zinc-induced ROS generation in breast epithelial carcinoma cells (51). When damage is severe or irreparable, p53 can promote apoptosis by regulating pro-apoptotic and anti-apoptotic proteins like Bax and Bcl-2, with Bcl-2 confirmed to have a central role in the intrinsic apoptosis pathway (52). Liu and colleagues further confirmed in CuSO4-treated ICR mice that high-dose Cu^2+^ exposure induces oxidative stress response by increasing ROS and protein carbonyl compound (PC) levels, downregulating antioxidant superoxide anion (ASA), anti-hydroxyl radical (AHR), superoxide dismutase (SOD), catalase (CAT), and glutathione peroxidase (GSH-Px) activities, and reducing glutathione (GSH) content and mRNA expression levels (31).

Copper ion carriers produce reactive oxygen species upon binding to copper, inducing oxidative stress, damaging DNA, and causing cell cycle arrest (53). Shimada et al. demonstrated that NSC319726 can act as a carrier for Zn^2+^ and Cu^2+^, exerting effects in various cancer cell models. Its binding activates and promotes redox reactions between Cu^2+^ and Cu^+^, resulting in the depletion of deoxyribonucleotides, DNA synthesis inhibition, and G1 phase cell cycle arrest, ultimately triggering apoptosis (54). Yip et al. found that disulfiram (DSF) exhibits strong copper-dependent toxicity *in vitro* against cultured breast cancer cells. The combined application of disulfiram and Cu^2+^ can inhibit breast cancer cell colony formation and significantly enhance paclitaxel cytotoxicity. The specific mechanism involves the disulfiram-Cu^2+^ complex inducing ROS production, subsequently activating downstream apoptosis-related JNK and p38MAPK pathways while inhibiting the NF-kB signaling pathway, thereby inducing breast cancer cell apoptosis. This suggests that the combination of copper ions and anticancer drugs generates ROS, forms oxidative stress, suppresses anti-apoptotic factors, activates apoptosis-related pathways, and ultimately induces cancer cell apoptosis (55). Likewise, copper transport proteins and chaperone proteins, such as ATP7A, ATP7B, Atox1, and CCS, strictly regulate intracellular copper homeostasis (56–58). Dysregulation of these proteins may lead to abnormal intracellular copper distribution, thereby affecting apoptotic signaling pathways. For example, mutations in ATP7B result in Wilson’s disease, characterized by copper accumulation in the liver and brain, causing cell death and tissue damage (59).

**Figure 3:**
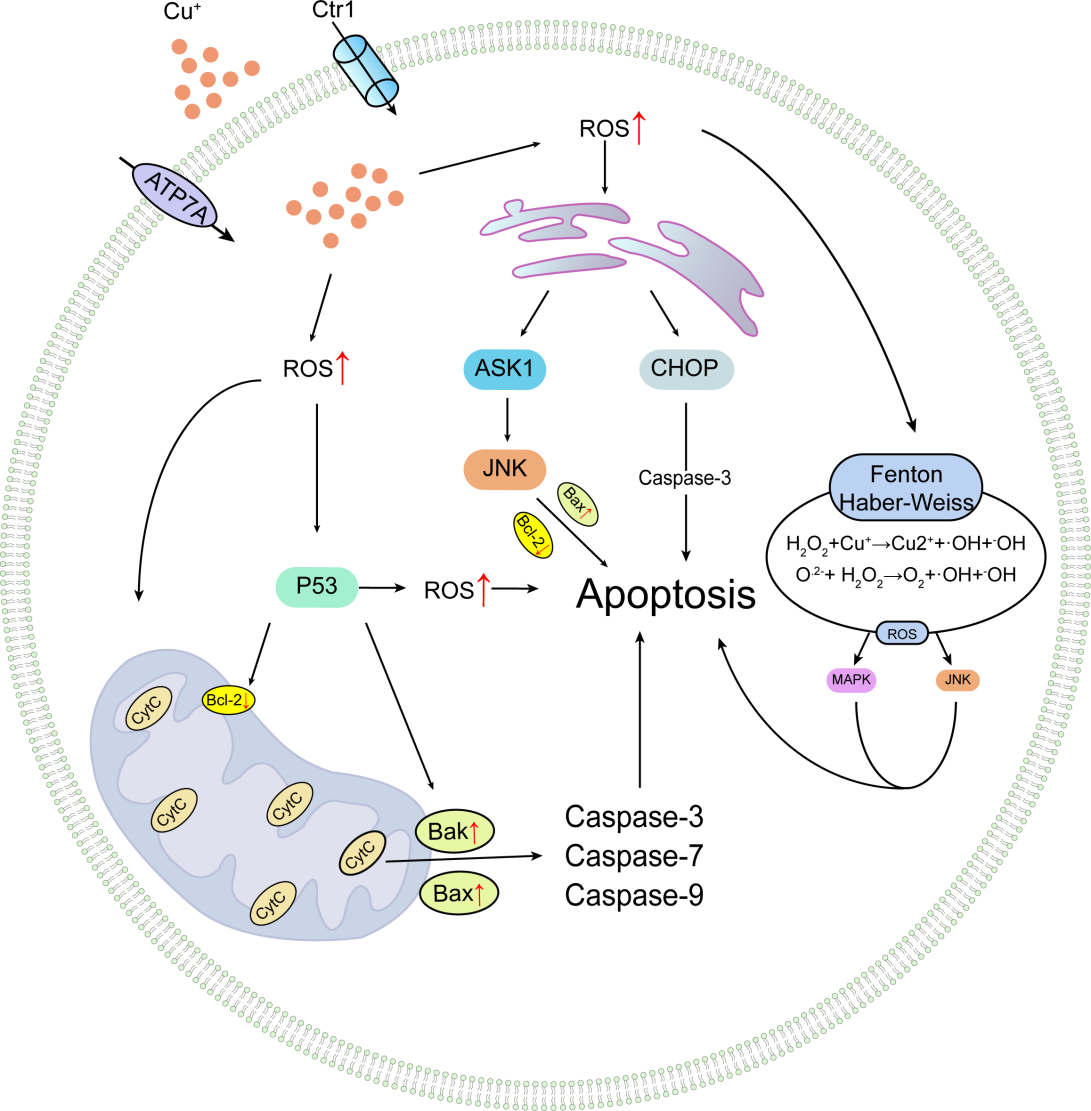
Conceptual illustration of copper-induced apoptotic processes.

As previously mentioned, copper is a crucial factor in the functional regulation and maintenance of stability for various enzymes, some of which play roles in apoptotic pathways. For instance, cytochrome c oxidase is of paramount importance for mitochondrial function maintenance; impairment of cytochrome c oxidase can lead to mitochondrial dysfunction, alterations in mitochondrial membrane permeability, and ensuing mitochondrial dysregulation. Copper can also damage the mitochondrial electron transport chain and inhibit ATP synthesis, further exacerbating cellular energy metabolism disorders, potentially triggering the activation of apoptotic signaling pathways and culminating in cell death (8). In prior experiments by Liu and colleagues, high-dose copper exposure induced hepatocyte apoptosis through the mitochondrial apoptosis pathway, leading to mitochondrial membrane depolarization, release of cytochrome c, cleavage of caspase-9 and caspase-3, increased levels of Bak and Bax, decreased Bcl-2, and ultimately inducing apoptosis (31). Additionally, copper-derived copper complexes can promote cancer cell apoptosis. Research by Cen and colleagues revealed that Cu^2+^ and disulfiram form a complex, bis(diethyldithiocarbamate)copper [Cu(deDTC)], through intricate extracellular redox reactions, unique to Cu^2+^. This copper complex exhibits heightened anti-melanoma activity, causing a significant increase in melanoma cell line apoptosis (60). Wu et al. assessed the impact of copper on endoplasmic reticulum stress and hepatocyte apoptosis in ICR mice treated with copper sulfate (CuSO4). The results demonstrated that CuSO4 significantly induced hepatocyte apoptosis and endoplasmic reticulum stress (markedly increased mRNA and protein levels of glucose-regulated protein 78 (GRP78) and 94 (GRP94)). Moreover, in CuSO4-treated mice, elevated intracellular calcium activated three apoptosis pathways triggered by endoplasmic reticulum stress, resulting in increased mRNA and protein levels of CHOP, JNK, and caspase-12 pathways in liver cells, activating corresponding pathways and augmenting hepatocyte apoptosis (47), as illustrated in **Figure 3**.

### 3.2 Copper-induced cellular necrosis

Cellular necrosis represents another form of cell death, characterized by the disruption of normal cellular metabolism due to various causes, leading to morphological alterations and culminating in cell lysis and destruction, a process known as necrosis (10). This process incites an inflammatory response and inflicts damage on surrounding tissues. Necrosis is typically considered an accidental, unregulated form of cell death, rather than the highly regulated apoptotic process (61).

As previously mentioned, copper can participate in redox reactions, generating reactive oxygen species (ROS), such as superoxide and hydroxyl radicals (62, 63). When ROS production surpasses the cell’s antioxidative defenses, oxidative stress ensues, damaging cellular components such as DNA, proteins, and lipids. Under severe oxidative stress, damage to cellular structures, including the plasma membrane, leads to cell necrosis (44). Imbalance in intracellular copper homeostasis, with abnormally high concentrations, may induce toxic reactions, impair the function of copper transport proteins and chaperones, and result in cell necrosis. Excessive intracellular copper can damage organelles and the plasma membrane, ultimately causing cell necrosis. Copper ions can also interact with the lipid bilayer of the plasma membrane, rendering it unstable (64). This interaction may lead to lipid peroxidation and membrane integrity disruption, ultimately resulting in cell necrosis. Furthermore, copper-induced oxidative stress can exacerbate membrane damage through promoting lipid peroxidation (65, 66).

### 3.3 Copper-induced cellular autophagy

Copper-induced autophagy is an intricate biological mechanism, whereby cells stimulate an autophagic response to counteract excessive internal copper. This process aids in maintaining physiological equilibrium by eliminating redundant or dysfunctional components within the cellular structure (67). Under circumstances where there’s a surplus of copper in the cellular environment, cells initiate autophagy as a self-defensive mechanism.

Empirical studies suggest that exposure to elevated copper levels can instigate an autophagic response in certain cell types.Though the precise mechanics of copper-induced autophagy are not yet wholly understood, several probable pathways have been proposed. One such potential mechanism operates through oxidative stress. Copper can trigger the production of reactive oxygen species (ROS), which subsequently impair proteins, lipids, and DNA, leading to heightened cellular stress. This stress, in turn, can incite an autophagic response, employed by the cell as a means to eradicate damaged components and maintain cellular homeostasis (10). An alternative possible mechanism might operate via direct interaction with proteins involved in the autophagic pathway. A number of studies suggest that copper can bind to these proteins and alter their activity, thus precipitating the autophagic process (68).

Copper could also potentially influence autophagy via its role in various signaling pathways (69). For instance, research has established that mTOR (mammalian target of rapamycin) is a key regulatory factor in autophagy (70), and that copper can trigger an autophagic response by influencing the activity of mTOR. For example, when mTOR’s activity is impeded, it induces the occurrence of autophagy (71). Furthermore, Luo and colleagues discovered that mtROS can mediate the Akt/AMPK/mTOR pathway, thereby participating in copper-induced autophagy in RAW264.7 mouse monocytes and inhibiting copper-induced apoptosis (72).

### 3.4 Copper-induced pyroptosis

Pyroptosis, also known as inflammatory necrosis, is a lytic form of programmed cell death characterized by continuous cellular swelling until the cell membrane ruptures, resulting in the release of cellular contents. This process not only clears damaged cells but also elicits a potent inflammatory response (73, 74). Pyroptosis is typically triggered by the recognition of pathogen-associated molecular patterns (PAMPs) or damage-associated molecular patterns (DAMPs) in response to microbial infection or cellular stress.

The occurrence of pyroptosis relies on the activation of certain caspase family proteins by inflammasomes. Caspase-1 is an enzyme responsible for the maturation and secretion of pro-inflammatory cytokines, such as interleukin-1β (IL-1β) and interleukin-18 (IL-18). Inflammasome activation leads to the cleavage and activation of caspase-1, which in turn cleaves gasdermin D (GSDMD). Activated gasdermin proteins translocate to the membrane, forming pores that cause cellular swelling, cytoplasmic leakage, and ultimately, cell membrane rupture and pyroptosis (75, 76). The pores formed by GSDMD permit the release of pro-inflammatory cytokines, ultimately resulting in cellular swelling, membrane rupture, and cell death (77). Research has shown that pyroptosis is extensively involved in the development of infectious diseases (78), neurodegenerative disorders (79) and atherosclerotic conditions (80, 81), playing a critical role in their progression. In-depth study of pyroptosis may help elucidate its role in the onset, progression, and outcome of related diseases, providing novel insights for clinical prevention and treatment (82, 83).

Some studies have demonstrated that copper exposure activates NLRP3 inflammasomes, resulting in pyroptosis-mediated neurotoxicity. Researchers, such as Dcigcndcsch et al., employed a mouse model to explore the *in vivo* effects of copper consumption on NLRP3-dependent inflammation. Their findings revealed that intracellular copper ions are key factors in NLRP3 inflammasome assembly and subsequent activation of cytokines such as caspase-1 and IL-1β. They also discovered that copper depletion specifically blocks classical NLRP3-mediated pyroptosis due to the downregulation of copper-dependent SOD1 activity. *In vitro* experiments with copper chelator TTM-treated human bone marrow-derived cells showed that monocyte IL-1β secretion and pyroptosis were insensitive to copper depletion. However, peritoneal macrophages isolated from non-infectious ascites patients treated with lipopolysaccharide and nigericin, followed by TTM copper chelation, exhibited reduced IL-1β secretion, suggesting that classical NLRP3 activation in macrophages is subject to specific regulation by copper homeostasis (84). These findings imply that copper mediates macrophage pyroptosis and participates in the regulation of inflammatory responses through the NLRP3 inflammasome activation pathway, indicating that targeting intracellular copper homeostasis may represent a potential therapeutic approach for NLRP3-dependent diseases. Tao et al. utilized copper oxide nanoparticles (CuONPs) to treat murine macrophages and observed increased levels of proteins and mRNA, such as NLRP3, Caspase-1, and IL-1β, as well as increased IL-1β release. Administration of NLRP3 siRNA and Z-YVAD-FMK reduced the expression of CuONPs-induced Caspase-1 p20 and IL-1β, suggesting that CuONPs promote NLRP3-dependent pyroptosis in macrophages via the NF-κB pathway. The study revealed that CuONPs activation of NLRP3 inflammasomes is a dual process. On one hand, CuONPs attack induces lysosomal damage while releasing cathepsin B, which directly mediates NLRP3 inflammasome activation. On the other hand, following deposition in lysosomes, CuONPs may release copper ions due to the acidic environment of lysosomes.

**Figure 4:**
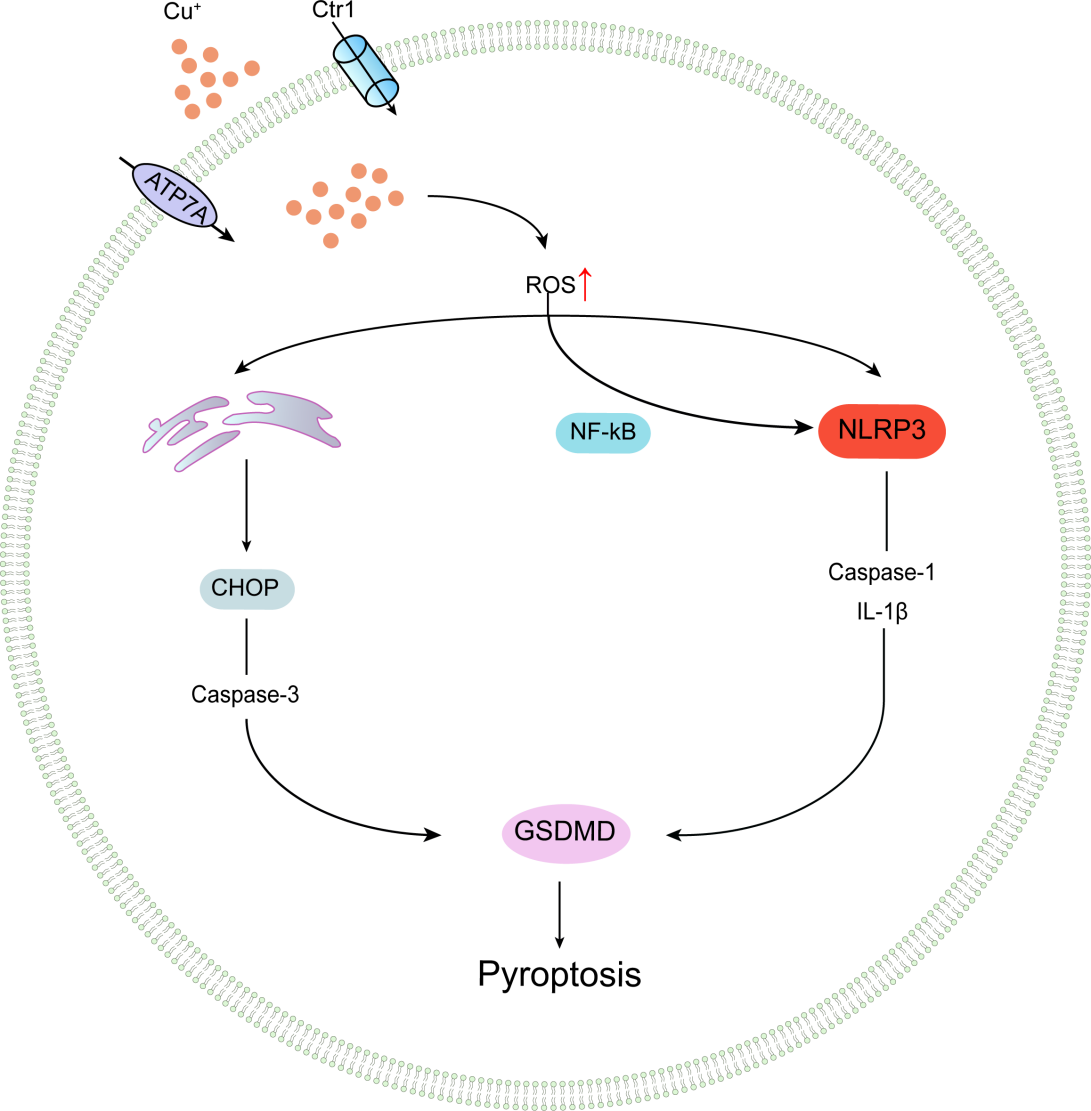
Conceptual representation of copper-elicited pyroptotic events.

Thus, the released copper ions significantly induce cellular oxidative stress and further mediate NLRP3 inflammasome activation, ultimately leading to NLRP3-dependent pyroptosis. Experiments incubating CuONPs in artificial lysosomal fluid revealed that acidic conditions release substantial amounts of Cu2+ ions, and H2O2 enhances the signal of hydroxyl adducts produced by CuONPs and copper ions, suggesting that copper ions cause oxidative stress through valence state transitions in Fenton or Haber-Weiss reactions, thereby inducing NLRP3 inflammasome activation through multiple cascading pathways. TTM pre-treatment reduces CuONPs-induced reactive oxygen species generation and NLRP3 activation, indicating that macrophage NLRP3-dependent pyroptosis requires copper involvement, and reducing copper availability can alleviate CuONPs-induced macrophage pyroptosis-mediated immunological damage (85).Recently, Dong et al. treated primary non-mutant control mouse-derived microglial cells with CuCl2 and lipopolysaccharide, observing time-dependent increases in NLRP3, cleaved Caspase-1, ASC, and IL-1β protein levels. This suggests that CuCl2 exposure triggers microglial cell NLRP3 activation and Caspase-1 levels, subsequently mediating inflammation and neurotoxic responses (86), as depicted in **Figure 4**.

### 3.5 Copper-induced cell death

#### 3.5.1 Cuprotosis

Tsvetkov et al.’s 2019 research discovered that during mitochondrial energy metabolism alterations, the process of oxidative phosphorylation increases tumor cells’ resistance to proteasome inhibitors, but also enhances sensitivity to the novel small molecule drug elesclomol (ES) (87). Elesclomol is a copper ion carrier, currently used as an anticancer drug, capable of forming complexes with Cu2+ ions. The elesclomol-Cu2+ complex is transported to mitochondria, where Cu2+ is reduced to Cu+ by ferredoxin 1 (FDX1), subsequently inducing ROS-dependent apoptosis and ultimately leading to cell death, with the specific mechanism yet to be elucidated (88). Caspase-3 and Caspase-7 are downstream effectors of the apoptotic pathway; elesclomol treatment does not activate the apoptosis marker caspase-3, and blocking the apoptotic pathway or other known programmed cell death pathways with inhibitors does not prevent copper-induced cell death, indicating that copper-induced cell death differs from known cell death modes.

In March 2022, Tsvetkov et al.’s research demonstrated that excessive intracellular copper induces the aggregation of dihydrolipoyllysine-residue acetyltransferase (DLAT), which is associated with the mitochondrial tricarboxylic acid (TCA) cycle, and is related to mitochondrial activity, resulting in a novel form of copper poisoning-induced cell death. For the first time, this novel copper-dependent death mode was named “Cuproptosis,” elevating the study of copper-induced cell death mechanisms to a new level (5). The research showed that abnormally elevated copper ions in human cells may induce cell death through a pathway distinct from known RCD mechanisms; inhibitors of apoptosis, necrosis, and ROS-induced cell death cannot prevent copper from continuing to induce cell death.

Cuproptosis occurrence depends on intracellular copper accumulation. Elesclomol (ES) treatment alone does not affect the growth of malignant rhabdomyosarcoma MON cells, but the addition of copper ions inhibits cell growth, while other metal ions (such as iron, cobalt, zinc, nickel, etc.) cannot enhance cell death. Depleting intracellular GSH with buthionine sulfoximine increases elesclomol (ES)-induced human hepatocellular carcinoma JHH7 cell death, whereas the copper chelator TTM combined with elesclomol (ES) treatment of human non-small cell lung cancer ABC1 cells does not affect their growth in the presence of elesclomol (ES), indicating that elesclomol participates in promoting a unique copper-dependent cell death, which cannot be blocked by apoptosis inhibitors (5).

**Figure 5:**
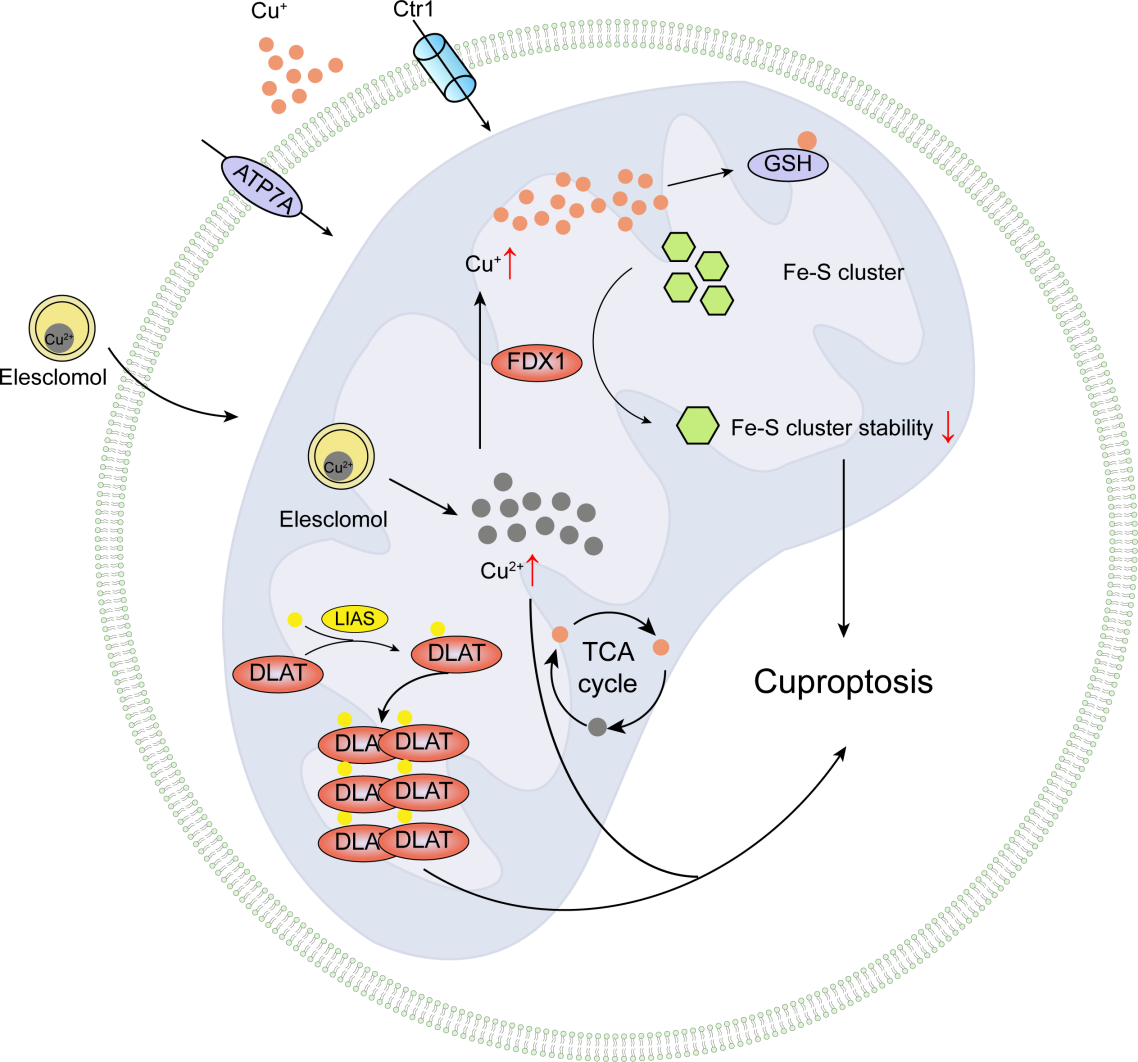
Pictorial delineation of cuproptotic processes.

The copper-induced demise involves a protein lipoylation process, a rare and highly conserved lysine post-translational modification, which has only been observed in four lipoylated protein complexes in mammals: pyruvate dehydrogenase (PDH), alpha-ketoglutarate dehydrogenase (KDH), branched-chain alpha-keto acid dehydrogenase (BCKDH), and the glycine cleavage system (GCV) (89). PDH and KDH directly participate in the tricarboxylic acid cycle, BCKDH is responsible for decarboxylation in branched-chain amino acid catabolism, and GCV plays a vital role in glycine breakdown (89, 90). These enzymes are essential for maintaining normal mitochondrial metabolism, either directly or indirectly participating in the mitochondrial tricarboxylic acid cycle, binding with copper ions, causing lipoylated protein aggregation, and subsequent iron-sulfur cluster protein loss, leading to mitochondrial metabolic dysfunction, proteotoxic stress, and ultimately cell death. The study results demonstrate for the first time that knocking out key upstream regulatory factors of lipoylated proteins, FDX1 or lipoylation-related enzymes, can block copper-induced cell death, indicating that FDX1 regulates DLAT lipoylation. Additionally, researchers show that copper promotes dihydrolipoamide S-acetyltransferase (DLAT) oligomerization, resulting in insoluble DLAT increase, further leading to proteotoxic stress and cell death, as depicted in **Figure 5**.

FDX1 and protein lipoylation are crucial factors in regulating copper-dependent cell death induced by ES. Protein lipoylation, a highly conserved lysine post-translational modification, attaches lipoic acid moieties to lysine residues of substrate proteins, modulating their functions. Protein lipoylation occurs exclusively in essential components of pyruvate dehydrogenase complexes, such as dihydrolipoamide S-succinyltransferase (DLST) and DLAT, where lipoylated enzyme modifications play a pivotal role in regulating and facilitating enzymatic functions (89).

Ferredoxin 1 (FDX1), as the core regulatory gene of copper-induced cell death, has been scarcely studied. In Xu et al.’s research, immunoinfiltration and single-cell analyses reveal the indispensable role of FDX1 expression in macrophages and monocytes. Tissue microarray analysis shows reduced FDX1 expression in tumor tissues of patients with renal clear cell carcinoma. Knockdown of FDX1 leads to decreased copper deposition in renal clear cell tumor cells. In renal clear cell tumor cells, FDX1-related gene expression features are associated with the enrichment of tricarboxylic acid (TCA) cycle, NOTCH pathway, and other related genes (91). In Tsvetkov et al.’s study, FDX1 and lipoylated protein expression are highly correlated in breast cancer and ovarian cancer samples. Furthermore, in FDX1-knockout human pancreatic cancer PSN1 cells, the expression of lipoylated proteins DLAT and DLST is completely lost, accompanied by a decline in cellular oxygen consumption rate. Additionally, metabolite analysis of FDX1-knockout human chronic myelogenous leukemia K562 cells reveals the accumulation of pyruvate and alpha-ketoglutarate in the lipoic acid metabolic pathway, suggesting inhibition of the tricarboxylic acid cycle. This confirms that FDX1 is an upstream regulatory factor of protein lipoylation, regulating cell copper death induced by ES via the tricarboxylic acid cycle (5).

Through an in-depth investigation into the intrinsic connection between copper-induced cell death and the TCA cycle, it has been discovered that copper-induced cell death is mediated by protein lipoylation and is closely related to mitochondrial activity. Research findings demonstrate that cells highly reliant on mitochondrial respiration are more sensitive to copper-induced cell death, suggesting a strong correlation with the tricarboxylic acid cycle (TCA). This may be associated with elevated levels of lipoylated proteins in cells with vigorous mitochondrial metabolism and an active TCA cycle, providing new perspectives and approaches for the treatment of hereditary copper overload disorders and cancer. Naturally, numerous questions still require further exploration in the study of copper-dependent cell death, such as the mechanisms by which FDX1 functions in the lipoylation process, the definitive markers for copper death, the regulatory mechanisms of protein degradation (e.g., autophagy and ubiquitin-proteasome system) in controlling copper death, targeted interventions and regulatory mechanisms for copper homeostasis disruption caused by exogenous substances, and how copper death initiates, propagates, and ultimately occurs.

#### 3.5.2 Copper-induced cellular mortality and its potential clinical applications

The elucidation of the mechanism underlying copper-induced cellular death delineates a promising avenue for future clinical interventions. Agents that induce cellular death by acting as copper ion carriers, also known as cell-death-associated drugs, could potentially hold significant promise in future cancer therapies (92, 93). Studies suggest that Disulfiram (DSF) can induce cellular death by relocating copper ions within cells and mitochondria (94), leading to a cascade of effects, including DLAT oligomerization, decreased Fe-S stability, and interaction with Npl4. Given the significant correlation between copper toxicity induced by these copper ion carriers and the level of mitochondrial metabolism, this insight could be harnessed in investigations of potential copper-toxicity-based treatments for certain tumours that inherently exhibit elevated levels of mitochondrial metabolism, such as melanomas, breast cancer, and leukaemia (23, 95). This association could be leveraged in future research exploring potential pharmaceuticals for cancer treatment based on copper toxicity (96). Moreover, cancer stem-like cells in certain cancers, such as glioblastoma (97, 98) and cholangiocarcinoma (99), also demonstrate higher levels of aerobic respiration. For instance, Elesclomol, once regarded as an oxidative stress inducer, has been found to inhibit cancer progression through copper-induced death, and its anticancer activity is contingent upon the cancer’s dependency on mitochondrial metabolism. Advancements in research into the relationship between Elesclomol, mitochondrial metabolism, and copper-induced death present the possibility of a renewed clinical application of Elesclomol.

Additionally, researchers are dedicated to studying new copper ion carriers as potential targeted cancer therapeutics (100, 101). Yang et al. have explored the feasibility of curcumin as an anticancer copper ion carrier. Their findings suggest that curcumin, acting as a copper ion carrier, can regulate lipid, RNA, NADH, and NADPH metabolism in colorectal cancer cells, thereby establishing a crucial link between copper-induced cell death and potential new anticancer agents based on this mechanism (102).

In summary, copper ion carriers, combined with targeted therapeutic agents, could be utilised for tumours characterised by high mitochondrial metabolism and high aerobic respiration, providing potential therapeutic directions for the combined use of copper-toxicity-inducing copper ion carriers and small molecule drugs in targeted treatment of specific tumours.

## 4 Copper complexes in anti-tumor processes

Research has elucidated that copper ions participate in epithelial-mesenchymal transition and the activation of signaling pathways such as Mitogen-Activated Protein Kinase (MAPK), further modulating cellular apoptosis, necrosis, autophagy, and prompting angiogenesis surrounding the tumor. This subsequently impacts the proliferation and metastasis of tumorous cells. It is currently posited that copper is a candidate metal component for anti-tumor pharmaceuticals.Given copper’s physiological characteristics and its potent ability for redox reactions, Cu+ and Cu2+ ions can interact with a multitude of ligands. Specifically, Cu+ ions can bind with diphosphine, nitrogenous ligands, and multiple pyridine compounds, while Cu2+ ions can bind with Schiff bases, disulfiram, and para-chlorobenzoic acid ligands, thus engendering a variety of distinctive copper complexes. The corresponding mechanisms of action related to cell death induced by Disulfiram-Copper binary complex, a ternary copper complex combined with 1-10-phenanthroline and L-tyrosine, and excess copper are delineated in **Table 1**.

**Table 1:** Pertinent mechanisms of action.

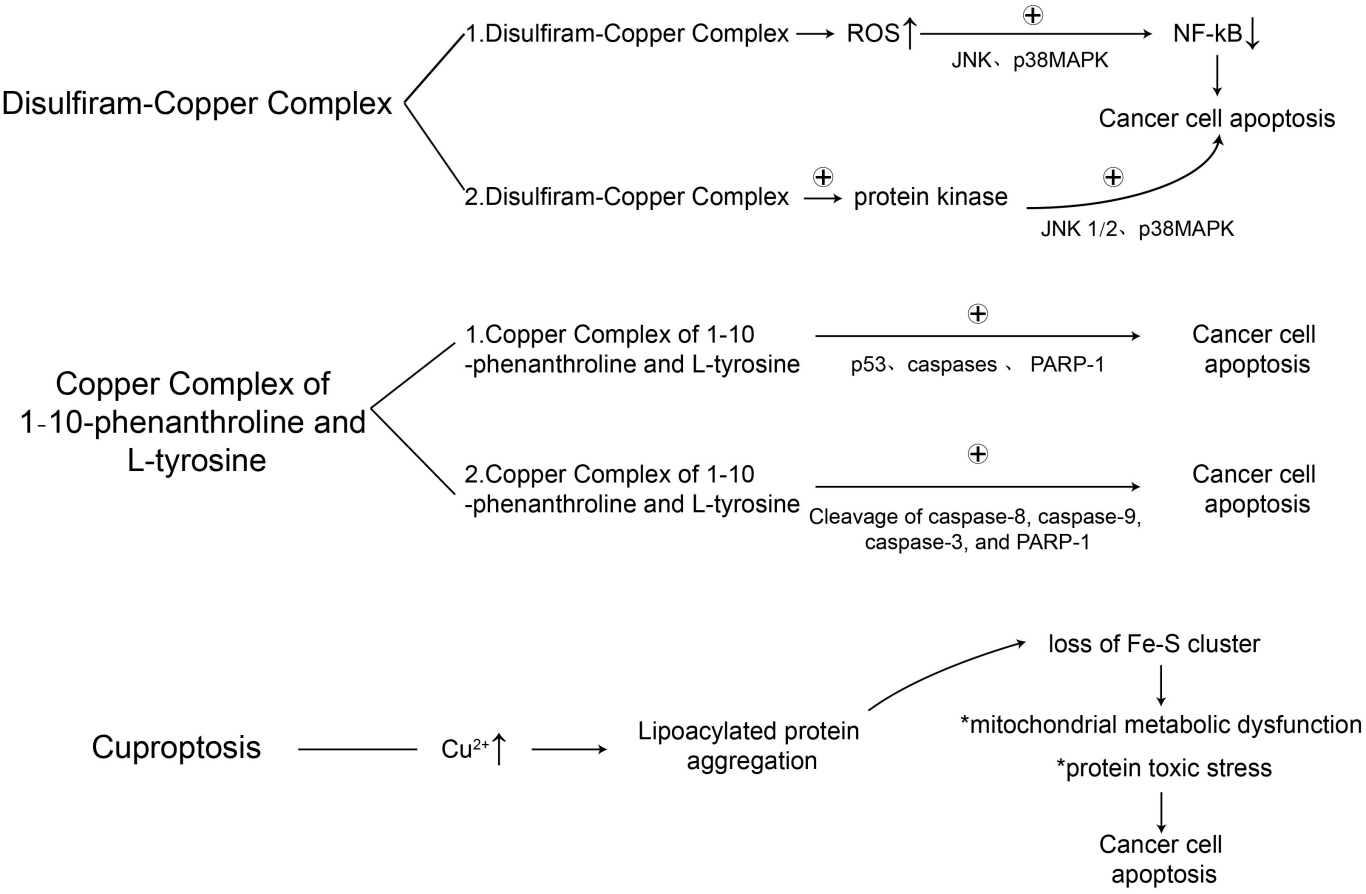

### 4.1 Binary copper complexes

Owing to their capacity to interact with biomolecules such as DNA, RNA, and proteins, coupled with their potential to induce oxidative stress within cancer cells, copper complexes have been recognized as promising candidates for anti-tumor therapeutics. Binary copper complexes refer to those containing copper and a single ligand. Over the past few years, numerous binary copper metal complexes have been discovered possessing cytotoxic or enzyme inhibitory functions, capable of inducing apoptosis in tumor cells (67, 103). Zhong and associates synthesized a copper complex using a novel Schiff base as the ligand. The *in vitro* anti-tumor activity experiments demonstrated that the new complex and its ligand exhibit a certain degree of inhibitory action against tumor cells (104). Existing research has confirmed that the complex formed by the Schiff base and copper can inhibit tumor growth, prevent tumor metastasis, and prolong the survival of tumor-bearing animals (105, 106). The complex formed by curcumin and copper can also decelerate the growth rate of animal transplant tumors. Studies have shown that curcumin and its derivatives can effectively combat triple-negative breast cancer (TNBC) cell lines *in vitro*, thus inhibiting the proliferation of these breast cancer cells (107). Carboxamidrazones complexed with copper can effectively inhibit the proliferation of murine melanoma B16F10 cells. The complex formed by divalent copper and carboxamidrazones can facilitate the intracellular transport of copper ions, inhibit the activity of estrogen receptor proteins, and also restrain the activity of cyclin-dependent kinases (108).

Disulfiram (DSF) is a therapeutic substance conventionally employed to combat alcohol intoxication. Recently, burgeoning research has unveiled its anti-carcinogenic properties and potential to augment the efficacy of tumor chemotherapy. Further studies revealed that the collaboration of DSF and copper could possibly manifest effective anti-carcinogenic characteristics.Reactive oxygen species (ROS) are requisite molecules in chemical and biological reactions, where moderate levels of ROS facilitate cellular proliferation and differentiation. However, an excess concentration of ROS may inflict substantial damage to cellular structures, including lipids, proteins, and DNA. This oxidative stress can lead to cell cycle arrest, autophagy, apoptosis, or even cell death - especially in cancer cells, which are often more susceptible to ROS-induced damage. Current research indicates that DSF/Cu complexes can generate ROS within cancer cells (109, 110). Guo et al. conducted a study on the toxicity of disulfiram/copper (DSF/Cu) complex both *in vitro* and *in vivo* against human osteosarcoma (OS). The results indicated that DSF/Cu could significantly trigger the generation of ROS, subsequently inducing cell cycle arrest, autophagy, and apoptosis in a ROS-dependent manner. Furthermore, the research demonstrated that DSF/Cu could notably inhibit the proliferation and clonal formation of OS cell lines. DSF/Cu primarily inhibits the growth of OS possibly by inducing apoptosis through the activation of the ROS/JNK pathway. In addition, DSF/Cu also inhibited the growth of OS in heterograft models with minimal organ-related toxicity (111). Similarly, Liu et al., while exploring the anti-tumor mechanism of disulfiram in gastric cancer (GC), found that low non-toxic concentrations of copper (Cu) ions significantly enhanced the anti-tumor activity of DSF, inhibiting the proliferation and growth of GC cell lines. DSF/Cu induced anti-tumor activity against gastric cancer via ROS/MAPK and NPL4 pathways, increased the production of reactive oxygen species (ROS), and induced apoptosis in a ROS-dependent manner (112). The ubiquitin-proteasome system (UPS) plays a critical role in maintaining protein metabolism equilibrium by degrading proteins such as cell cycle factors and regulatory proteins, thereby regulating numerous vital life activities and maintaining normal cell function. Chen et al. discovered that DSF-copper complex could effectively inhibit the proteasomal activity in cultured breast cancer MDA-MB-231 and MCF10DCIS.com cells before inducing apoptotic cancer cell death, but it did not inhibit the proteasomal activity in normal immortalized MCF-10A cells. Upon administering DSF to mice bearing MDA-MB-231 tumor xenografts, it significantly inhibited tumor growth through *in vivo* proteasome inhibition and induction of cell apoptosis. The study confirmed that by inhibiting proteasomal activity, targeting tumor cell copper with the non-toxic compound DSF could lead to selective induction of apoptosis within tumor cells (113). These results suggest that the DSF/Cu complex might be an effective and safe choice for treating OS in clinical settings. As the anti-cancer activity of DSF has been considered copper-dependent, Skrott et al. concluded from their comparison among four groups of mice injected with human MDA-MB-231 cancer cells that DSF/CuGlu treatment was superior to DSF treatment alone, with the DSF/CuGlu treatment group showing a tumor volume reduction of up to 77% by day 32. These results validated previous conclusions that DSF is an effective anticancer agent, and that copper enhances its activity.Subsequently, Skrott et al. continued to research the DTC-copper complex (diethyldithiocarbamate-copper (CuET)), and found that CuET inhibits the binding of p97 to the proteasome substrate linker NPL4, blocking the p97-dependent protein degradation and inducing cancer cell death.

In recent years, with in-depth research on nanomaterials, binary copper complexes and other nanomedicines have gradually demonstrated the potential to kill cancer cells with minimal invasiveness and high specificity. Ma et al. presented research on self-assembled copper-amino acid thiol nanoparticles (Cu-Cys NP) for *in-situ* glutathione activation and H2O2-enhanced chemodynamic therapy of drug-resistant breast cancer. Upon cellular uptake of Cu-Cys nanoparticles by tumor cells, they first react with local GSH, inducing GSH depletion and reducing Cu2+ to Cu+. Subsequently, the generated Cu+ reacts with local H2O2 to generate toxic hydroxyl radicals (·OH) through a Fenton-like reaction. This process has a rapid reaction rate in the mildly acidic TME, which is the main cause of induced tumor cell apoptosis (114).

### 4.2 Ternary copper complexes

Ternary copper complexes are composed of a copper ion and two different types of ligands. Similar to binary complexes, they have demonstrated potential as anticancer agents. These complexes are referred to as ‘ternary’ because they consist of three parts: the copper ion (Cu), the primary ligand, and the secondary ligand. The antitumor properties of ternary copper complexes enable them to generate reactive oxygen species (ROS), thereby causing cellular damage. In the case of cancer cells, this damage can result in cell death, slowing or preventing tumor growth. The exact mechanism of how ternary copper complexes exert their anticancer effects is not yet fully understood. However, current research indicates that, similar to binary copper complexes, they may act as anticancer agents through interactions with DNA and proteins within cancer cells (115).

Some ternary copper complexes can interact with mitochondria in tumor cells or normal cells, blocking the process of oxidative phosphorylation, inhibiting respiration, and showing antitumor activity against various tumor cells both *in vivo* and *in vitro* (116). In addition, ternary copper complexes can competitively bind with DNA, as well as binding with nucleases, thus inhibiting their binding with plasmid, genes, and DNA within the nucleosome, and inducing tumor cell apoptosis by generating oxygen free radicals (117). For instance, the ternary copper (II) complex combined with 1-10-phenanthroline and L-tyrosine displayed active anticancer effects in HT-29 colon cancer cells (118, 119). In Arikrishnan et al.’s experiment, the intracellular reactive oxygen species (ROS) was detected by DCFH-DA assay. The expressions of proteins involved in apoptotic signaling pathways (p53, caspases, and PARP-1) were assessed by Western blot analysis. The results showed that the ternary copper (II) complex reduced the cell viability of HT-29 cells in a time- and dose-dependent manner. Morphological assessment and membrane-associated protein V-FITC/PI flow cytometry analysis confirmed the cleavage and activation of caspase-8, caspase-9, caspase-3, and PARP-1, promoting cell apoptosis and leading to S-phase cell cycle arrest (120).

Indeed, like other potential anticancer drugs, the antitumor efficacy of ternary copper complexes heavily relies on their ability to specifically target cancer cells without affecting normal cells. The bioavailability, stability, and potential side effects of these compounds must also be thoroughly evaluated before clinical application. Furthermore, research is ongoing to better understand their mechanism of action and to develop more effective and safer copper-based anticancer drugs.

## 5 Applications of copper complexes in cancer treatment

Contemporary research predominantly focuses on the provision of copper, with copper-based nanomaterials, copper complexes, and nano-copper complexes all being utilized in oncological therapeutic investigations. With the advancement of nanotechnology, copper-based nano-drugs have garnered extensive attention in the realm of cancer therapy, serving not only as excellent drug carriers, but also demonstrating therapeutic effects directly. In tandem with the rise of nanotechnology, copper nanomaterials characterized by their diminutive size, superior biocompatibility, and capacity for tumor targeting have also begun to be deployed in cancer treatments (121). Given the tremendous success of cisplatin in cancer therapy, copper complexes are also being comprehensively examined as potential alternatives. As we deepen our understanding of copper complexes in the context of chemodynamic therapy, localized phototherapy for tumors, immunotherapy, and inducing immunogenic cell death, it offers an alternative perspective and strategy for cancer therapy.

### 5.1 Chemodynamic therapy

In recent years, chemodynamic therapy (CDT) has garnered widespread attention as an emerging treatment modality (122). CDT refers to the conversion of H2O2 generated in tumor tissue into highly oxidative hydroxyl radicals through metal ion-mediated Fenton or Fenton-like reactions, thereby killing tumor cells (123). Copper complexes play a crucial role in chemodynamic therapy as they can regulate the concentration of copper ions in the tumor microenvironment and induce tumor cell death by catalyzing the production of reactive oxygen species (ROS), leading to damage to biomacromolecules (124). In contrast to traditional anti-cancer treatments, CDT is an endogenous chemically activated therapy that does not require the introduction of anti-cancer drugs or external stimulation. With strong tumor specificity, outstanding targeting, minimal adverse reactions, and simple operation, CDT represents an emerging direction in cancer treatment. The rapid development of nanotechnology in recent years has enabled the widespread exploration of nanomaterials’ exceptional light, ultrasound, magnetic, and other stimulus-responsive properties, not only making it possible for CDT to synergize with other treatment methods in cancer therapy but also demonstrating superior anti-cancer activity compared to single treatments (125, 126).

Kordestani et al. reveal that employing raw materials such as copper nitrate, ethylenediamine, 3,5-dibromosalicylaldehyde, and triethylamine to synthesize Cu2+ complexes—namely 3,5-dibromosalicylaldehyde imine copper nitrate—suppresses the proliferation of human A2780 ovarian cancer cells (127). To enhance drug specificity for tumor cells, Luo et al. devised a feasible biotinylated copper complex, Bio-CuCl, which selectively targets biotin receptor-positive tumor cells, inflicting cancer cell-specific destruction (128). Nonetheless, poor penetrative abilities of nanoparticles and antioxidant activities of tumor cells diminish the efficacy of chemodynamic therapy (CDT). Consequently, Zheng et al. employed zinc protoporphyrin IX-loaded copper nanoparticles and polyethylene glycol-coated copper molybdenum oxide (CuMoOx) to inhibit heme oxygenase-1 activity, combine with GSH, and obstruct its antioxidative function, thus providing a favorable oxidizable microenvironment for CDT and bolstering its tumor cell-killing effect (129).

Schiff base metal complexes have garnered increasing attention for their capacity to influence DNA structure and function by forming hydrogen or other chemical bonds with DNA bases, as well as their unique biological properties. Zhao et al. studied five copper complexes with bis-Schiff base ligands, of which complexes 1-3 are mononuclear and 4-5 are dinuclear([Cu (L 1)] (1), [Cu (L 2)] (2), [Cu (L 3)] (3), [Cu 2 (L 4) (OAc)] (4), and [Cu 2 (L 5) (HCOO)] (5) bearing the bis-Schiff base ligands of bis (5-chlorosalicylidene) -1,3-propanediamine (H 2 L 1), bis (5-chlorosalicylidene) -2-methyl-1,3-propanediamine (H 2 L 2), bis (5-bromosalicylidene) -2-methyl-1,3-propanediamine (H 2 L 3), bis (5-chlorosalicylidene) -2-hydroxyl-1,3-propanediamine (H 3 L 4), and bis (5-bromosalicylidene) -2-hydroxyl-1,3-propanediamine (H 3 L 5), respectively). All five complexes demonstrated higher cytotoxicity against cancer and normal cell lines compared to cisplatin, with complex 2 exhibiting the most potent antitumor effect. Further research into the anticancer activity of the three mononuclear complexes against Hela cells revealed that they induced early apoptosis via the mitochondrial pathway, elevating intracellular ROS and Ca2+ levels while reducing mitochondrial membrane potential. Additionally, these complexes activated intracellular caspase-3 and caspase-9 and regulated the expression of pro-apoptotic and anti-apoptotic proteins. These results indicate that complex 2 may serve as a potential anticancer drug (130).

Research by Ghorbanpour et al. showed that copper(II) complexes with N,S-donor pyrazole-based ligands exhibited significant cytotoxicity against human breast cancer cell lines *in vitro*. These compounds also displayed considerable anticancer activity and properties (131). In comparison to free ligands, copper complexes exhibited enhanced interactions with EGFR and CDK2 proteins. Due to lower toxicity than other metal centers such as platinum, copper-based complexes have emerged as attractive candidates for further evaluation as anticancer agents. Consequently, copper complexes have garnered increased attention in cancer therapy and can be further assessed as chemotherapeutic drugs for cancer treatment.

Monotherapies often lead to the emergence of drug resistance in tumor cells, whereas synergistic treatments can enhance therapeutic efficacy. Chen and colleagues discovered that a cooperative starvation-CDT therapy generates a consistent supply of hydrogen peroxide through glucose oxidation, fueling Fenton reactions and producing more hydroxyl radicals. This collaboration achieves a synergistic, positive effect in inhibiting tumor growth. By employing sequential assembly, peanut-shaped nanoparticles are constructed by encapsulating CuS nanoparticles within a polypeptide-glucose oxidase-hyaluronic acid shell. The hyaluronic acid shell, with charge conversion and specific recognition for CD44, provides the copper nanomedicine with prolonged circulation time and tumor-targeting capabilities, as well as extended retention at the tumor site. Upon exposure to glucose oxidase, endogenous glucose is depleted for starvation therapy, followed by highly toxic hydroxyl radical generation via Cu2+-mediated Fenton-like reactions for CDT (132). Additionally, these nanoparticles recruit various immune cells for anti-tumor immunotherapy. Cu2+ and DNA nuclease combine to form a mixed nanomaterial, cooperatively transported into tumor cells. Cu2+ is reduced to Cu+ by GSH for CDT, while DNA nuclease cleaves RNA, effectively targeting vascular endothelial growth factor-2 (VEGF2) mRNA and downregulating VEGF2 gene expression for gene therapy. The coordinated anti-tumor effect induced by the Cu2+ and DNA nuclease complex effectively inhibits *in vivo* tumor growth (133). Many unresolved issues remain in the chemodynamic therapy of copper complexes, such as complex selection, dosage, and potential toxicity. Future research must further investigate these issues to provide more effective and safer treatment options for clinical practice

### 5.2 Local phototherapy for tumors

Local phototherapy is an emerging non-invasive cancer treatment that effectively inhibits tumor growth, primarily including photodynamic therapy (PDT) and photothermal therapy (PTT) (134, 135). PDT relies on the illumination of a specific wavelength light source to activate photosensitizers in tumor tissue, generating biotoxic singlet oxygen and other reactive oxygen species. This leads to oxidative damage to tumors, virus-infected cells, and other overproliferating cells, activating anti-tumor and anti-viral immunity, damaging blood vessels, and killing bacteria, fungi, and viruses to eliminate inflammation (136, 137). The use of photosensitizers is a key component of PDT, while PTT can enhance treatment efficiency and efficacy without exogenous photothermal agents (138). In phototherapy, light intensity is spatially controlled, selectively targeting tumor cells (139). PDT employs tissue-penetrating photosensitizers, which are injected into the body. Due to the high absorption and low metabolism characteristics of tumor tissue, photosensitizers can specifically accumulate within tumors, then activated by a specific wavelength laser, catalyzing a series of chemical reactions to produce reactive oxygen species, ultimately inducing tumor cell death (140).

In recent years, researchers have demonstrated keen interest in photodynamic therapy. Wang and colleagues found that novel copper complexes formed by carboxyl groups and copper ions, known as copper-doped carbon dots (Cu-CDs), can generate robust photo-induced cytotoxicity, effectively inhibiting the growth of human cervical cancer and neuroblastoma cells (141). This suggests that copper-doped carbon dots may be a promising photosensitizing agent. Shrestha and colleagues discovered that copper cysteamine nanoparticles, as novel photosensitizers, can significantly reduce the volume of subcutaneously implanted right mammary tumor tissue in mice upon X-ray radiation activation, potentially serving as an excellent candidate for deep tumor photothermal therapy (142). Zhang et al. treated *in-situ* malignant glioblastoma using a secondary near-infrared (NIR-II) photodynamic therapy and chemotherapy with ultrasmall Cu2-xSe nanoparticles. It was found that ultrasmall Cu2-xSe NPs exhibit strong absorption characteristics in the NIR-II window, and their potent NIR-II absorption and deeper tissue penetration of NIR-II light ensure outstanding photodynamic therapeutic performance. Ultrasmall Cu2-xSe nanoparticles generate abundant reactive oxygen species via electron transfer. Additionally, these nanoparticles can be effectively delivered to *in-situ* malignant glioblastoma tissue, assisting in focused ultrasound. The deposited Cu2-xSe nanoparticles can also be utilized for photoacoustic imaging to guide the combination therapy of NIR-II photodynamic therapy and chemotherapy. The results indicate significant tumor growth inhibition, demonstrating the immense potential of drug-loaded ultrasmall Cu2-xSe nanoparticles as a promising therapeutic agent for treating *in-situ* malignant glioblastoma (143). Xu and colleagues developed a glucose oxidase (GOx)-engineered porous copper(I) 1,2,4-triazole ([Cu(tz)]) coordination polymer (CP) nanoplatfom, denoted as GOx@[Cu(tz)], for starvation-enhanced copper poisoning and photodynamic synergistic therapy (144). Consumption of glucose and GSH renders cancer cells highly susceptible to GOx@[Cu(tz)]-mediated copper poisoning, inducing a massive accumulation of acylated mitochondrial proteins. Glucose oxidation elevates intracellular hydrogen peroxide (H2O2) levels, activating the Type I photodynamic therapy (PDT) efficacy of GOx@[Cu(tz)]. *In vivo* experiments show that GOx@[Cu(tz)] inhibits tumor growth by 92.4% with minimal systemic toxicity, aiding researchers in further exploring effective copper poisoning-based cancer treatment strategies.

In addition to photothermal therapy, photodynamic therapy is also prevalent, achieving localized hyperthermia through the utilization of photothermal materials with near-infrared absorption under laser irradiation, effectively eradicating tumor cells. Hou and colleagues discovered that transferrin-modified mesoporous hollow copper sulfide nanoparticles exhibit prolonged accumulation and retention at the site of breast cancer tumors in tumor-bearing mice, demonstrating potent near-infrared absorption and photothermal conversion efficiency. They effectively convert near-infrared light into thermal energy for photothermal treatment while generating high levels of reactive oxygen species for photodynamic therapy (145). Weng and colleagues prepared a novel photothermal agent—copper nanoparticles loaded in carbon polyhedra—under different temperatures in an argon atmosphere. Intravenous injection of these nanoparticles, followed by 808 nm wavelength light irradiation, caused a rapid temperature increase at the subcutaneously implanted cervical cancer tumor site in mice within 3 minutes. The final temperature reached 64.6°C after 10 minutes, achieving effective thermotherapy results, and complete tumor tissue regression after 20 days. Conversely, intravenous injection of PBS in mice showed no significant temperature changes in tumor tissue under continuous light exposure, and the tumor volume increased considerably after 20 days (146). This suggests that the nanomaterial exhibits favorable near-infrared light absorption characteristics, high photothermal conversion efficiency, and potential applications for anti-tumor treatment.

Combined therapies have garnered widespread interest in mitigating the shortcomings of single treatments and enhancing therapeutic efficacy. Pertaining to such research, Liu and colleagues investigated Tween-20 (Tw 20) modified and doxorubicin (Dox) loaded Cu2S nanoparticles (Cu2S/Dox@Tw20 nanoparticles), which significantly improved tumor treatment performance. The drug loading capacity in Tw 20 increased, as did colloidal stability and biocompatibility. Due to the Dox loading, Cu2S/Dox@Tw20 nanoparticles exhibited chemotherapeutic activity, with a tumor inhibition rate of 76.2%. Further combined with near-infrared laser treatment, elevated temperatures directly induced extensive tumor cell apoptosis, while concomitantly released chemotherapy drugs under heating conditions not only continued to eradicate residual tumor cells but also inhibited tumor recurrence. Consequently, tumors were completely eliminated under the combination of photothermal therapy (PTT) and chemotherapy (147). Zhang et al. found that hollow mesoporous silica nanoparticles (HMSNs) functioned as nanocarriers in the delivery of Cu2+-doped polydopamine (PDA), termed HMSNs@PDA-Cu, facilitating synergistic treatment. PDA, as a traditional photothermal agent, enabled photothermal treatment (PTT). Upon coordination with Cu2+, PDA’s photothermal properties improved while also exhibiting superoxide dismutase (SOD) activity. PDA converted O2·- (O2·-) into H2O2, increasing H2O2 production and further promoting chemodynamic therapy (CDT) efficacy (148). Moreover, the elevated temperatures induced by PTT further enhanced CDT’s ·OH production rate, suggesting tremendous potential in *in vitro* and *in vivo* cancer metastasis treatment. Xu and colleagues proposed a novel copper-chelated polydopamine nanosystem (Cu-PDA-FA) with surface polyethylene glycol modification and folic acid (FA) targeting. As a photothermal agent (PTA), Fenton-like reaction initiator, and “immunogenic cell death” inducer, it mediated PTT/CDT synergistic tumor therapy and anti-tumor immune activation, offering new insights into constructing a universal nanoplatform for tumor treatment (149). Despite the immense potential of copper complexes in localized phototherapy for tumors, several issues remain to be addressed, such as the selection of the most effective copper complexes, determining optimal light irradiation dosages and timing, and evaluating potential toxicity and safety. More clinical trials are required in the future to assess the safety and efficacy of copper complexes in localized tumor phototherapy.

### 5.3 Immunotherapy

Tumor immunotherapy is a treatment modality that harnesses the human immune system to effectively recognize and attack cancer cells, combating malignancies (150). Immune checkpoint inhibitors are among the most prevalent immunotherapeutic approaches, whereby they suppress immune checkpoint molecules on tumor cell surfaces, thus activating the host immune system and intensifying the assault on neoplasms, ultimately achieving therapeutic aims (151, 152). Anti-programmed death protein 1 (PD-1) and programmed death-ligand 1 (PD-L1) are two critical immune checkpoint proteins (153). Tumor cells expressing PD-L1 can bind to the PD-1 receptor on T lymphocytes, inhibiting the cytotoxic T cells’ capacity to destroy tumor cells and consequently causing immune suppression (154). Disulfiram (DSF), as a potential chemosensitizer and anticancer drug, has been proven to kill tumor cells by regulating multiple signaling pathways, transcription factors, and through the accumulation of copper ions and inhibition of proteasome activity in cancer cells. It also possesses antiangiogenic properties and induces epigenetic modifications. Zheng et al. demonstrate that DSF increases PD-L1 expression in triple-negative breast cancer (TNBC) cells. *In vivo* experiments indicate that DSF significantly improved the response to anti-PD-1 antibodies (Ab) in a 4T1 breast cancer mouse model, suggesting that the combination of DSF and anti-PD-1 antibodies can activate the tumor immune microenvironment, exhibiting far superior antitumor efficacy compared to monotherapy (155).

In recent years, studies have confirmed that copper can modulate PD-L1 expression. Voli and colleagues demonstrated that an increase in CTR1 expression in neoplastic cells led to elevated Cu2+ levels, activating epidermal growth factor receptor signaling pathways, which in turn increased PD-L1 mRNA and protein expression levels. When mice with neuroblastoma xenograft models were administered copper chelator tetrathiomolybdate, Cu2+ levels decreased, neuroblastoma cell PD-L1 expression was reduced, tumor-infiltrating T cell numbers increased, tumor growth slowed, and mouse survival rates improved. These findings suggest that diminishing copper ion concentrations within tumors through pharmaceutical intervention can enhance antitumor immunotherapy and imply that anticancer immunotherapy may be intensified by pharmacologically reducing copper levels within malignancies (156).

In the contemporary research milieu, there is an escalating number of investigations dedicated to the synthesis of chemotherapeutic and immunotherapeutic approaches. Immunotherapy leverages the intrinsic immune system of the patient to engender efficacious therapeutic responses, while chemotherapy holds the potential to overturn the immunosuppressive microenvironment of the tumor, thereby amplifying immunogenic effects and fostering anti-tumor reactions. The existing research findings underscore the synergistic potential of combined treatments.

Immunogenic Cell Death (ICD) embodies a specific variant of Regulated Cell Death (RCD), which constitutes a unique modality of cellular demise, capable of galvanizing the immune system to purge deceased cells and instigate an immune response against identical cells. This phenomenon holds particular significance in the realm of cancer treatment, whereby its characteristics can be harnessed to induce a cascade of immune response actions against tumor cells, thereby manifesting anti-tumor efficacy.

Recently, the research group led by Passeri reported the first metal-based drug 1, a copper (II) compound connected by bidentate 4,7-diphenyl-1,10-phenanthroline and tridentate Schiff base ligands, which efficiently eradicates Cancer Stem Cells (CSCs) through cytotoxic and immunogenic mechanisms (157). Studies revealed that the copper compound could escalate the intracellular levels of Reactive Oxygen Species (ROS), precipitating oxidative stress. This strain can trigger cell death, a form of demise typically characterized by immunogenicity. Furthermore, the copper complex disrupts the intracellular mechanisms responsible for protein synthesis, folding, and transport, inducing endoplasmic reticulum stress and yielding damage-associated molecular patterns (DAMPs) signaling molecules consistent with immunogenic cell death. These DAMPs are recognized by the immune system, further igniting the immune response.

Current research implies that insufficient T-cell infiltration significantly impedes the therapeutic efficacy of cancer immunotherapy. Inducing Immunogenic Cell Death (ICD) emerges as a promising strategy. It effectively releases tumor-associated antigens and damage-associated molecular patterns (DAMPs), which are captured by surrounding immune cells such as dendritic cells (DCs). Concurrently, it enhances the antigenicity of the tumor and the phagocytosis, maturation, and migration of dendritic cells (DC). Once activated, dendritic cells proficiently present tumor antigens to T cells, prompting their activation, proliferation, and infiltration, thereby initiating *de novo* T-cell immune responses. In essence, ICD, by liberating DAMPs, triggers antigen presentation, activates T cells, thereby fostering T-cell infiltration, which culminates in an effective anti-tumor immune response, directly attacking and annihilating tumor cells.

Wang and colleagues constructed a copper-based nanoscale coordination polymer (Cu-NCPs) with mixed valence states (Cu+/Cu2+). This compound can simultaneously and independently induce the generation of hydroxyl radicals triggered by Cu+ and the elimination of GSH triggered by Cu2+, effectively inducing ICD to synergize radiation therapy. This synergistic therapy significantly augments the maturation of dendritic cells and promotes the infiltration of anti-tumor CD8+ T cells, thereby enhancing an effective anti-tumor immune response and bolstering checkpoint blockade immunotherapy against primary and metastatic tumors (158).

Contemporary studies suggest that the combination of Disulfiram (DSF) and Copper (Cu) exhibits anti-tumor effects on a series of malignancies, including Hepatocellular Carcinoma (HCC). With chemotherapy drugs gradually discovered to enhance anti-tumor immunity by inducing Immunogenic Cell Death (ICD), Gao and his team explored the potential of DSF/Cu as an ICD inducer and its ability to enhance the efficacy of immune checkpoint blockade in HCC. The report showed that HCC cells treated with DSF/Cu exhibited ICD characteristics *in vitro*, such as Calreticulin (CRT) exposure, ATP secretion, and High Mobility Group Box 1 (HMGB1) release. HCC cells treated with DSF/Cu incited significant immune memory in vaccine inoculation assays while promoting dendritic cell activation and maturation. It was subsequently found that the combination of DSF/Cu and CD47 blockade further encouraged DC maturation and significantly enhanced CD8+ T cell infiltration, thereby promoting an effective anti-tumor immune response (159).

Cisplatin is known to be a first-line chemotherapeutic agent widely used in the treatment of various cancers. It is reported to promote mitochondrial ROS production (160) and induce endoplasmic reticulum (ER) stress and non-nuclear-dependent apoptosis (161). Compared with other chemotherapeutic agents that induce ER stress effects and promote ICD, the cell death induced by cisplatin is always silent. Ding et al. studied transforming immune tolerance into immunogenic cell death to enhance the therapeutic effect of cisplatin. Ding’s team developed ROS-sensitive nanoparticles loaded with copper chaperone inhibitor DC_AC50 and cisplatin (IV) prodrug. The released DC_AC50 can promote significant intracellular accumulation of cisplatin and copper by inhibiting the ATOX1-ATPase pathway, thereby enhancing the chemotherapeutic effect of cisplatin and inducing significant ROS production. Excessive ROS causes strong endoplasmic reticulum (ER) stress, thereby promoting ICD and triggering a persistent immune response. The study showed that copper chaperone inhibition mediated by DC_AC50 can restore the immunogenicity of tumor cells and is used to enhance chemotherapy and tumor immunotherapy.

In recent years, with the development of novel ICD-inducing metal complexes, ICD inducers that carry out therapeutic interventions by triggering local cell death in cancer cells and triggering an immune response throughout the organism have received widespread attention. ICD inducers as a new generation of anti-cancer agents have great prospects, can overcome the limitations of currently used drugs, and therefore can be applied to treat challenging and distant/metastatic tumors.

## 6 Conclusions

Copper, an essential trace metal, plays a pivotal role in maintaining normal hematopoietic function, promoting connective tissue formation, and facilitating cellular metabolism within the human body (162). Intricate balances of copper ion concentrations are maintained, and excess or deficiency can result in various maladies (163). Copper can induce cell death through multiple mechanisms, such as eliciting oxidative stress responses, inhibiting proteasomes, and through the recently discovered and designated “Cuproptosis” which promotes copper-targeted antitumor therapies (5). At present, two primary mechanisms have been identified for copper homeostasis in antitumor treatments: one involves copper chelators, which bind to and reduce copper ions, consequently suppressing tumor cell proliferation and metastasis; the other utilizes copper ion carriers to transport copper ions into cells, elevating intracellular Cu2+ levels, generating reactive oxygen species, and inducing tumor cell death (164). Further high-quality basic and translational research is needed to reveal the causal relationships between copper metabolism and multiple aspects of tumor cell and tumor microenvironment remodeling.

Elevated copper levels in tumor tissues and serum have been confirmed, indicating a strong correlation between copper ions and tumor development, rendering copper ions an attractive target for the development of novel cancer treatments. Advanced nanotechnology, which has emerged over the past several decades, offers immense potential for cancer therapy, and the applications of copper-based nanotherapeutic systems and copper complexes in antitumor treatments are garnering increasing attention (25). Copper complexes have been proven to synergistically interact with conventional chemotherapy and radiotherapy, enhancing anticancer efficacy. Currently, efforts are being made to investigate more efficient methods for precisely detecting and monitoring copper ions in the body, better classifying different types of copper coordination compounds, and determining their anticancer activities. Furthermore, it is essential to develop novel copper-dependent biomarkers to identify the most suitable populations for copper therapy, enabling personalized treatment strategies and facilitating clinical activity assessments.

## Author contributions

LG wrote the manuscript. AZ revised the manuscript. All authors contributed to the article and approved the submitted version.
