# Supplementary material for: Copper-instigated modulatory cell mortality mechanisms and progress in oncological treatment investigations
Source: Front Immunol. 2023 Aug 2;14:1236063. doi: 10.3389/fimmu.2023.1236063 (PMC10433393; doi:10.3389/fimmu.2023.1236063)
Supplement: Supplementary file 1 [file Image_1.pdf]

Supplementary Material

Copper-Instigated Modulatory Cell Mortality Mechanisms and Progress in Oncological Treatment Investigations

Lei Gao*

*** Correspondence:** Anqi Zhang: laughing_zhang1230@163.com

1 Supplementary Figures and Tables

1.1 Supplementary Figures

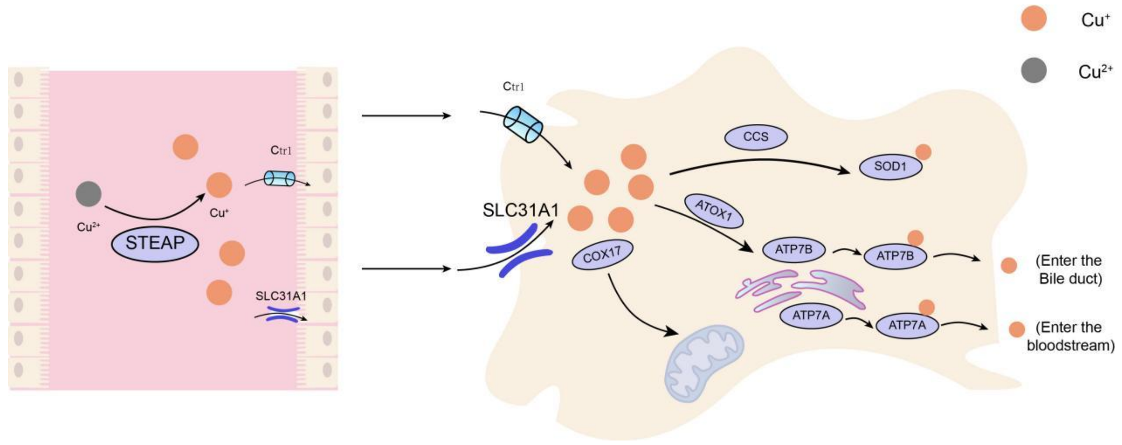

Supplementary Figure 1. The principal metabolic pathway of copper within cellular structures.

Notes: Cu, copper; Ctr1, copper transporter 1； CCS, copper chaperone for superoxide dismutase; SOD1, superoxide dismutase 1; COX17, cytochrome c oxidase copper chaperone 17; SLC31A1, Human High affinity copper uptake protein 1; ATOX1, antioxidant protein 1; ATP7A, ATP-dependent copper transporter 7A; ATP7B, ATP-dependent copper transporter 7B.

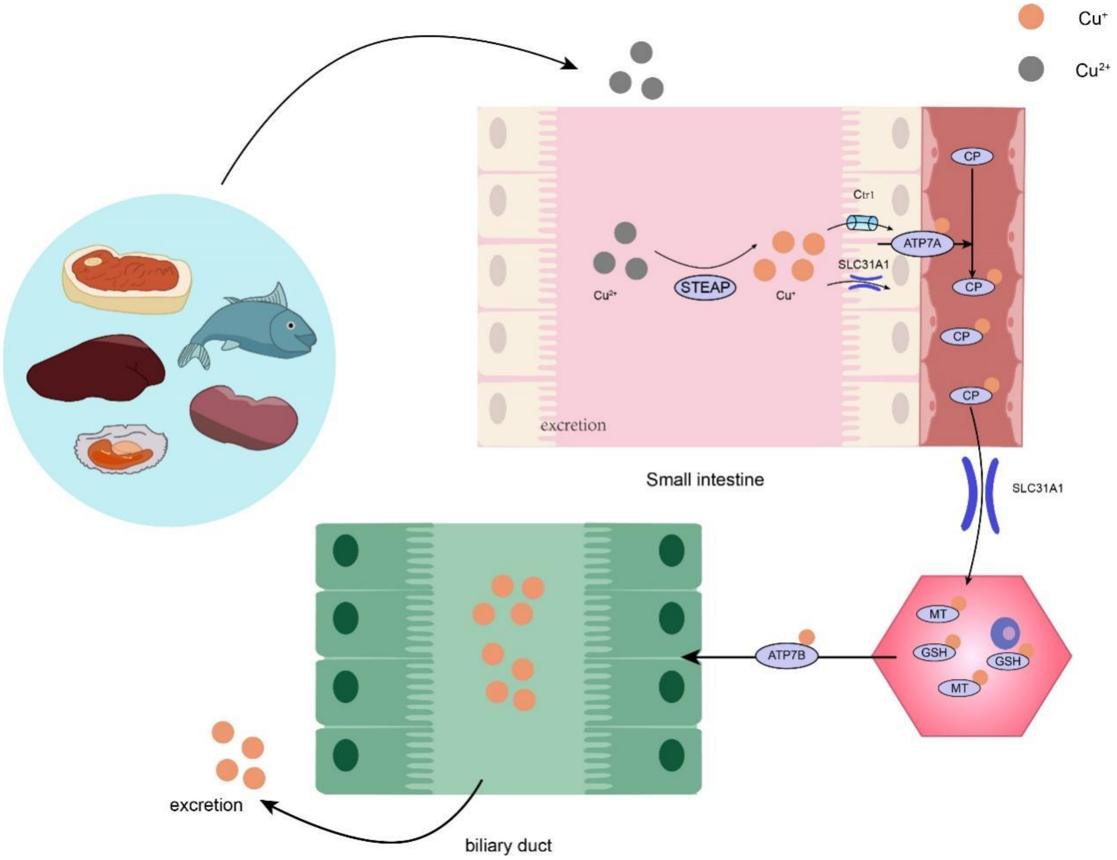

Supplementary Figure 2. The intricate procedure of copper metabolism within the human organism.

Notes: STEAP, STEAP Protein Family; CP, ceruloplasmin; SLC31A1, Human High affinity copper uptake protein 1; GSH, glutathione; MT, metallothionein.

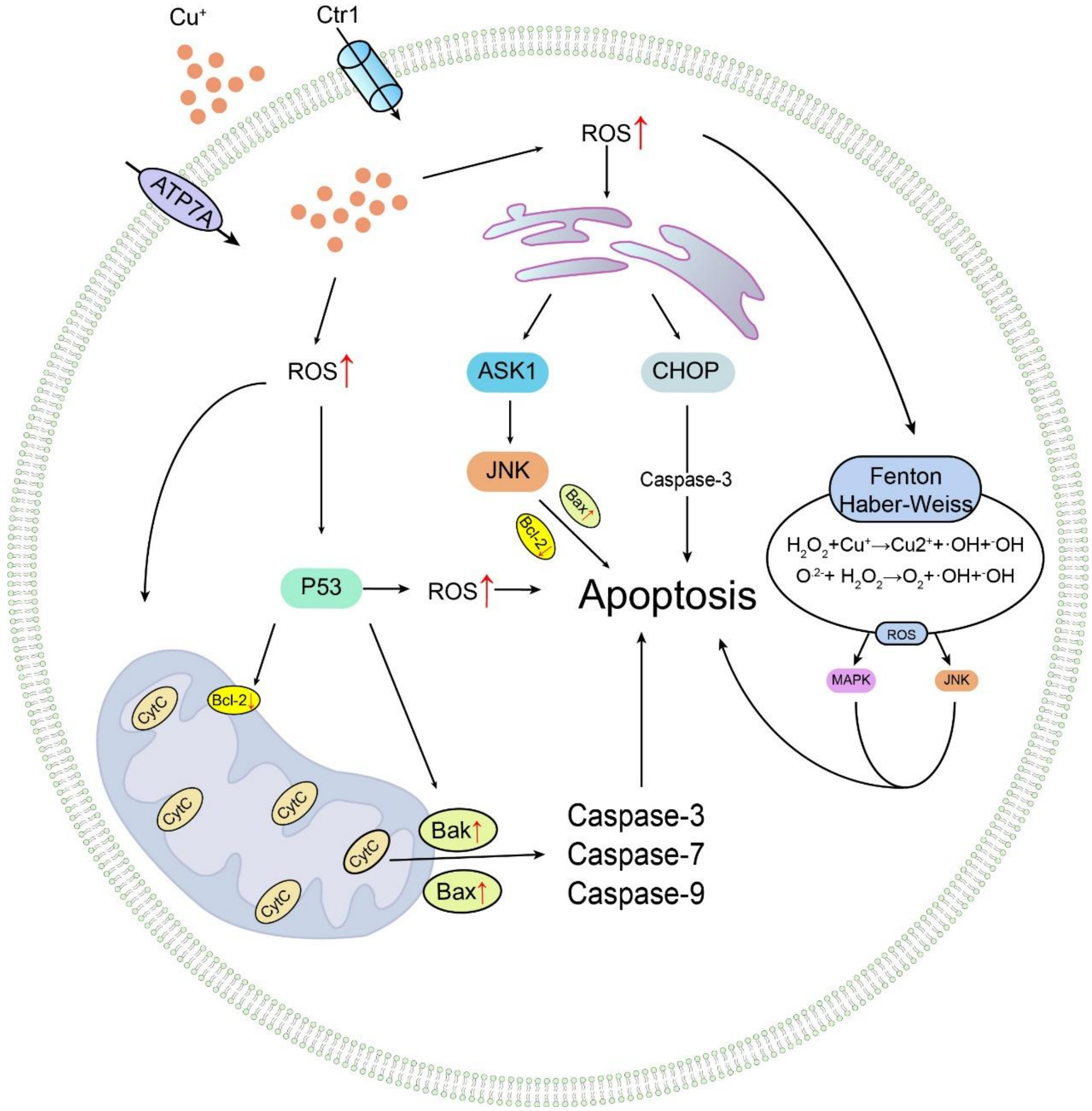

Supplementary Figure 3. Conceptual illustration of copper-induced apoptotic processes.

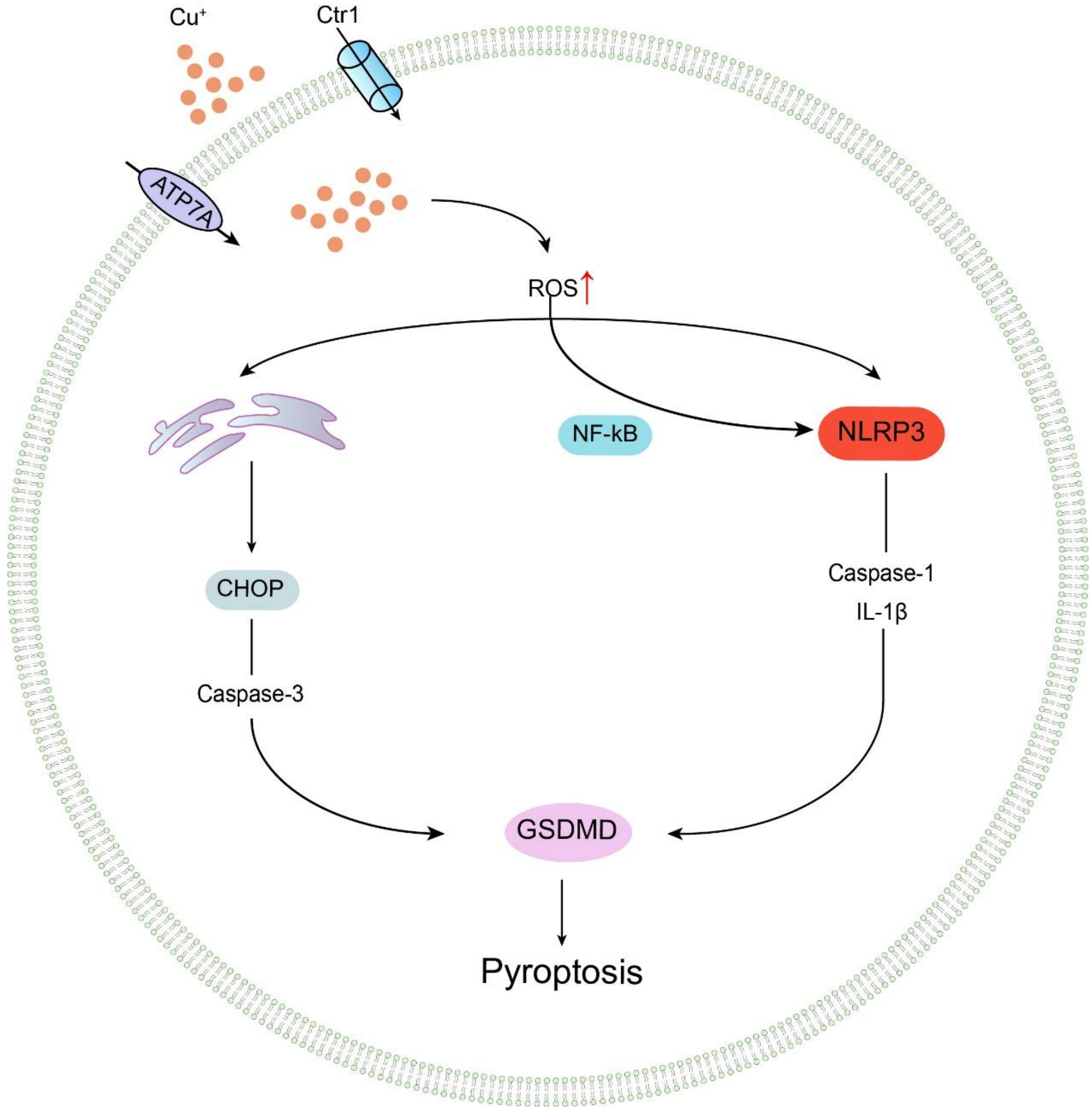

Supplementary Figure 4. Conceptual representation of copper-elicited pyroptotic events.

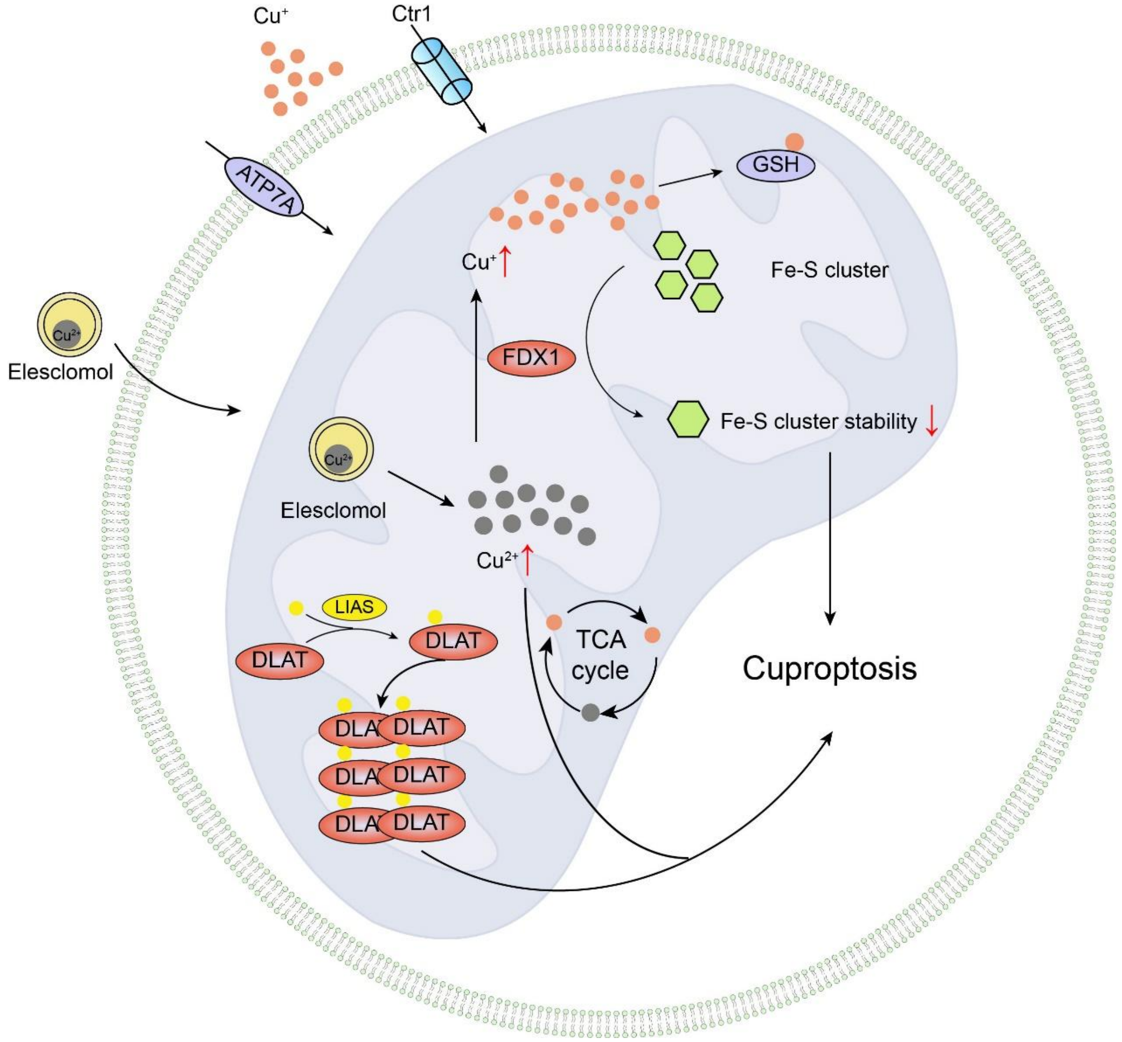

Supplementary Figure 5. Pictorial delineation of cuproptotic processes.
